# The Complexity of Mitochondrial Complex IV: An Update of Cytochrome *c* Oxidase Biogenesis in Plants

**DOI:** 10.3390/ijms19030662

**Published:** 2018-02-27

**Authors:** Natanael Mansilla, Sofia Racca, Diana E. Gras, Daniel H. Gonzalez, Elina Welchen

**Affiliations:** Instituto de Agrobiotecnología del Litoral (CONICET-UNL), Cátedra de Biología Celular y Molecular, Facultad de Bioquímica y Ciencias Biológicas, Universidad Nacional del Litoral, 3000 Santa Fe, Argentina; nmansilla@fbcb.unl.edu.ar (N.M.); sofia.racca@hotmail.com (S.R.); grasdiana@gmail.com (D.E.G.)

**Keywords:** mETC, OXPHOS, COX, plant growth, biogenesis

## Abstract

Mitochondrial respiration is an energy producing process that involves the coordinated action of several protein complexes embedded in the inner membrane to finally produce ATP. Complex IV or Cytochrome *c* Oxidase (COX) is the last electron acceptor of the respiratory chain, involved in the reduction of O_2_ to H_2_O. COX is a multimeric complex formed by multiple structural subunits encoded in two different genomes, prosthetic groups (heme *a* and heme *a*_3_), and metallic centers (Cu_A_ and Cu_B_). Tens of accessory proteins are required for mitochondrial RNA processing, synthesis and delivery of prosthetic groups and metallic centers, and for the final assembly of subunits to build a functional complex. In this review, we perform a comparative analysis of COX composition and biogenesis factors in yeast, mammals and plants. We also describe possible external and internal factors controlling the expression of structural proteins and assembly factors at the transcriptional and post-translational levels, and the effect of deficiencies in different steps of COX biogenesis to infer the role of COX in different aspects of plant development. We conclude that COX assembly in plants has conserved and specific features, probably due to the incorporation of a different set of subunits during evolution.

## 1. Introduction

Mitochondrial respiration is responsible for energy production in most eukaryotic organisms. The respiratory chain is composed of several multiprotein complexes attached to the inner mitochondrial membrane (IMM), which are involved in the transfer of electrons and the translocation of H^+^ for ATP synthesis through oxidative phosphorylation (OXPHOS). The respiratory complexes also contain prosthetic groups, which are essential for the electron transfer reactions. To establish a functional respiratory complex, several steps must be orderly followed, starting with the transcription of genes in two different compartments (the nucleus and mitochondria), the editing and processing of transcripts synthesized in the organelle, the synthesis, membrane translocation and assembly of the structural subunits that will finally conform the multiprotein complex and the synthesis and insertion of the prosthetic groups. All these steps must be finely controlled and are critical to ensure the assembly of a functional complex for the successful operation of mitochondrial respiration [1].

One of the respiratory complexes, complex IV or cytochrome *c* oxidase (COX), catalyzes the transfer of electrons from reduced cytochrome *c* (CYT*c*) to the final acceptor of electrons, O_2_, in a process that is coupled to H^+^ translocation for ATP production. Among the evidence that highlights the importance of the assembly process involved in COX biogenesis and function, one can mention the existence of more than 30 proteins specifically responsible for the synthesis and delivery of redox-active metal centers and prosthetic groups [2], and for the insertion and maintenance of properly assembled subunits into the IMM [3,4,5], the existence of multiple tissue- and growth condition-specific isoforms, and the existence of a significant number of severe human diseases connected to COX dysfunction [6,7]. In plants, multiple editing proteins and RNA-processing factors (pentatricopeptide repeat protein (PPR), RNA-editing factor interacting/ Multiple organellar RNA editing factor proteins (RIP/MORF), Organelle RNA recognition motif protein (ORRM) and the Organelle zinc finger editing factor family proteins (OZ)) [8,9] are required for proper expression of subunits encoded in mitochondria. In addition, mitochondrial respiratory activity must be connected to the energy requirements of the cells and those imposed by the environment. Thus, the COX assembly process must be regulated by internal and external factors in order to ensure the generation of the necessary energy through the mitochondrial OXPHOS pathway. The lack of mutant plants in COX components, due to embryonic lethality, highlight the importance of COX activity in plants and poses difficulties to the study of the assembly process. In this review, we summarize current knowledge about the COX assembly process and its regulation in plants, with references to other model systems in which the process has been more thoroughly characterized.

## 2. COX Biogenesis: from Yeast, to Mammals, to Plants

COX is present in mitochondria and also in several groups of prokarytotes, pointing to an endosymbiotic origin of the mitochondrial enzyme [10]. Bacterial COX is mainly composed of three subunits that form the catalytic core: COX1, COX2 and COX3. The eukaryotic enzyme is much more complex, with about 10 to 14 additional subunits that were most likely added after endosymbiosis [11,12]. The prokaryotic origin of COX is also reflected by the fact that the catalytic-core subunits are encoded in the mitochondrial genome in almost all organisms, with only a few exceptions, notably that of COX2, which is encoded in the nuclear genome in some leguminous plants [13]. The additional subunits are universally encoded in the nucleus and their evolutionary origin is not clear [3].

In addition to its polypeptidic components, COX contains redox cofactors involved in electron transfer reactions. A di-copper center (Cu_A_), present in COX2, receives electrons from reduced CYT*c* [14]. These electrons are then transferred to a heme *a* group present in COX1, to a binuclear heme *a*_3_-Cu_B_ center located in the same subunit, and finally to O_2_. Assembly of these redox cofactors requires the participation of a set of proteins which are conserved in prokaryotic and eukaryotic COX containing organisms. These are COX10 and COX15, involved in heme A synthesis [15,16,17], COX11 and SCO (Synthesis of Cytochrome *c* Oxidase) [18,19,20], involved in copper insertion, and SURF1 (Surfeit1), the role of which is not completely clear but may be related to heme A insertion into COX1 [21,22]. In parallel with the increased complexity of the eukaryotic enzyme, mitochondrial COX assembly requires a multitude of additional factors, about 30 described up to now. COX biogenesis has been widely studied in mammals and yeast (*Saccharomyces cerevisiae*). Because of the advantages of using *S. cerevisiae* as a model and the possibility of isolating respiratory-deficient mutants in this organism, studies in yeast pioneered the identification of proteins required for COX assembly [4,5,11,23]. The assembly process has been also extensively characterized in humans and other mammalian systems, due to the relevance of mitochondrial disorders in human diseases. In this sense, several revisions were recently published [5,6,7,11,12]. In the next paragraphs, we will briefly describe what is known about the COX assembly process in yeast and mammalian systems.

Current evidence suggests that COX is assembled from different modules, which are preassembled around the catalytic-core subunits [4,24,25]. COX1 is embedded in the IMM and contains 12 membrane-spanning domains. In yeast, it is inserted into the membrane during its synthesis with the help of Oxa1, Cox14 and Coa3. While Oxa1 has a more general role in the insertion of proteins in the IMM, Cox14 and Coa3 are COX1 chaperones that specifically aid in COX assembly and remain bound to COX1 after its insertion into the membrane. This complex also contains Mss51 and the Hsp70 chaperone Ssc1, involved in the feedback regulation of COX1 translation [26]. In humans, CMC1 (C-X9-C motif containing 1), instead of Mss51 and Hsp70, would be added to the complex to provide stability to the assembly intermediate [27]. Once in the membrane, heme A is added to COX1. Mss51 and Hsp70, as well as CMC1 in humans, probably exit the complex during this process, while the assemblies factor Coa1 and the structural subunits Cox5a and Cox6 are incorporated [28]. Heme A is synthesized from heme B in a two-step process catalyzed by the IMM enzymes Cox10 (heme O synthase) and Cox15 (heme A synthase). Due to its reactivity, it is assumed that heme is never released into the medium and is always bound to proteins [4]. However, how heme A is delivered to COX1 is not known. One possibility is that Cox15 directly handles heme A to COX1. Cox15 action requires its oligomerization and this is promoted by Pet117, which may aid in heme delivery to COX1 [29]. Another protein, SURF1, has also been involved in heme A insertion [21]. SURF1, named Shy1 in yeast, incorporates to the assembly complex at the time of heme A addition. The protein from the bacterium *Paracoccus denitrificans* is able to bind heme A, leading to the proposal that it functions as a heme chaperone during heme A insertion. However, mutation of a conserved histidine putatively involved in heme A binding in the yeast protein does not affect COX assembly. Alternatively, SURF1 may participate in heme A insertion as a COX1 chaperone, and it is possible that its function has diverged during evolution. This is also supported by the fact that SURF1 is not essential for COX assembly, but only to optimize the process [30]. Assuming that it is unlikely that heme A insertion occurs spontaneously, the actual factor directly responsible for this process remains unknown.

Assembly of the Cu_B_ center also takes place in the membrane after Mss51 release. Its relationship with heme A insertion is not known. Copper insertion is affected by Cox11, a membrane bound protein with a globular domain located in the intermembrane space (IMS) [31]. The Cox11 globular domain contains two conserved cysteines involved in copper binding. A third conserved cysteine is required for copper insertion into COX1, but not for copper binding by Cox11. This cysteine, in its reduced state, would participate in the transfer of copper from Cox11 to COX1 [32]. Another protein, Cox19, is required to maintain the reduced state of this conserved cysteine [33]. Cox19 is a copper-binding protein from the IMS. It has a twin CX_9_C motif found in several IMS proteins, many of which are related to COX assembly. Even if Cox19 was shown to bind copper, it is not involved in copper delivery to Cox11. This function is performed by another IMS twin CX_9_C protein, Cox17 [34,35]. In addition to the twin CX_9_C motif, Cox17 contains a CCXC copper-binding site that partially overlaps with the first CX_9_C. The source of copper for Cox17 is unknown. After proposals that Cox17 could translocate from the cytoplasm to the IMS with bound copper, the general consensus is currently that copper reaches the IMS bound to a low-molecular-weight ligand of unknown structure, and that the source of copper for Cox17 metalation is the mitochondrial matrix [36]. Accordingly, two IMM transporters, Pic2 and Mrs3, phosphate and iron carriers, respectively, were found to be able to transport copper into the matrix [37].

Cox17 also participates in the delivery of copper for the assembly of the Cu_A_ center in COX2. In this case, copper is transferred to Sco1, a protein with a similar location to Cox11. Sco1 and other SCO proteins have two conserved cysteines located in a CX_3_C motif, involved in copper binding together with a distal histidine [34,38]. With variations in different organisms, SCO proteins were implicated in the reduction of the copper-binding cysteines located in COX2 and/or the direct transfer of copper to COX2 (see [2] for details). Another protein from the IMS, Coa6, also participates in copper delivery to COX2, but its exact role is not clear at present [39].

Before copper insertion, COX2 must be correctly inserted into the IMM. COX2 has two membrane-spanning domains and a globular domain located in the IMS, where the Cu_A_ center is located. Thus, translocation of this domain from the site of synthesis, the matrix, to the IMS is also required. Membrane insertion is initiated by Oxa1 and the COX2-specific chaperone Cox20 [27,40]. During this process, an N-terminal extension involved in COX2 translocation is cleaved by the IMM protease Imp1 [41]. Then, Cox18, an Oxa1-like protein, together with Mss2 and Pnt1, promote the translocation of the globular domain through the IMM [40]. In humans, the COX2 module would also contain COX5B, COX6C, COX7B, COX7C and COX8A before assembling to COX1 and additional subunits [5,42]. Finally, COX3 would be incorporated to the complex. COX3 has been found in assembly intermediates containing Cox4, Cox7 and Cox13 in yeast, and COX6A, COX6B and COX7A, equivalent to yeast Cox13, Cox12 and Cox7, in humans [4,42].

It is evident that the COX assembly process has species-specific variations. This is reflected by the fact that certain assembly factors are present in some species but not in others. In addition, when putative homologs are present, they do not necessarily have similar roles. OXA1L, the human homolog of yeast Oxa1, for example, does not seem to be involved in COX1 or COX2 membrane translocation, as its counterpart in yeast [43]. In another example, yeast and humans have two SCO proteins, named 1 and 2. However, only Sco1 is essential for COX assembly in yeast, while both SCO1 and SCO2 have specific, non-redundant functions in COX assembly in humans [38,40,44]. In addition, the presence of alternative isoforms of certain subunits indicates that different forms of COX, probably with different properties, may coexist in the same organism or even in the same organelle [6,7]. Finally, COX may be found as a monomer, a dimer, or a component of supercomplexes, in association with other respiratory complexes. COX subunit composition varies in these different assemblies, suggesting that the incorporation of certain subunits modulates the formation of these structures. It has been proposed that the non-essential subunits Cox12 and Cox13 are involved in the formation of COX dimers. Efficient incorporation of Cox12 and Cox13 to COX requires Rcf1, a yeast protein involved in the accumulation of supercomplexes [45], suggesting that Cox12 and/or Cox13 may also participate in the incorporation of COX into supercomplexes [5]. Current views assume that the formation of dimers and supercomplexes changes the properties of the enzyme and may be used to modulate energy production according to cellular needs [46,47,48]. How these processes are regulated and whether COX assembly dynamically responds to these cellular needs is currently unknown.

## 3. COX Biogenesis Proteins in Plants

COX biogenesis in plants has not been extensively studied and most proteins associated with its structure and assembly were annotated by sequence homology. Trying to shed light on this scenario, we searched for genes encoding putative homologs of yeast and mammalian COX subunits or biogenesis proteins in the genome of the model plant *Arabidopsis thaliana*. As starting point, we used the information recently reviewed by Timón-Gómez et.al. [5] and Kadenbach [12]. We completed the list of candidates with the yeast proteins compiled by several authors [4,11,49,50,51,52,53,54] or by using a “keyword search” in the yeast genome database (yeastgenome.org; [55]). As an inclusion criterion, we only considered the yeast proteins for which experimental evidence about its biological connection with respiratory Complex IV was available. As a result, a total of 73 proteins from yeast and 62 candidates from mammals were included in the list. Of these, 26 are specific to yeast, five are unique to mammals, and 44 proteins are shared by the two organisms (see Table 1).

Using this dataset, we performed a bioinformatics analysis in the Phytozome 12 database (https://phytozome.jgi.doe.gov/pz/portal.html, [56]) to identify putative homologous proteins in the *Arabidopsis thaliana* genome. After this, experimental or predicted mitochondrial localization was corroborated for these candidate proteins (SUBA4, http://suba.live/, [57]; ARAMEMNON, http://aramemnon.uni-koeln.de/, [58]; Complexome, https://complexomemap.de/at_mito_leaves/, [59]). A total of 72 genes encoding putative *Arabidopsis* homologous COX-related proteins were found, which sum to 10 for already reported plant-specific COX subunits [59,60] (Table 1 and Table S1). From these, 4 encode the highly conserved structural catalytic-core subunits, usually encoded in the mitochondrial genome [61]. For COX3, an extra copy in the nuclear chromosome 2 is also present (Table 1), which is 100% identical to the mitochondrial gene. This region of chromosome 2, of around 600 kbp, presents hybridization with probes of mitochondrial DNA [62]. Nuclear *COX3* genes seem to be present also in other *Brassicaceae*, but it is not clear if they arise from the same DNA transfer event. To evaluate this, we performed a synteny analysis for the nuclear Arabidopsis *COX3* gene. Using Genomicus (Version 16.03, http://www.genomicus.biologie.ens.fr/genomicus-plants-16.03/cgi-bin/search.pl; [63]) and Plant Genome Duplication Database (PGDD; http://chibba.agtec.uga.edu/duplication/index/locus; [64]) databases, we observed that the gene order observed in *A. thaliana* is not conserved in other *Brassicaceae*, not even in *A. lyrata*. For comparison, other nuclear genes, such as At*COX10* or At*HCC2*, show gene arrangement conservation in all *Brassicaceae* (Figure S1a). This suggests that the Arabidopsis nuclear *COX3* gene is the result of a recent DNA transfer from the mitochondrion to the nucleus in *A. thaliana*. The region of 30 kbp that contains the nuclear *COX3* gene presents 15 additional genes, most of them almost identical at the sequence level with genes present in mitochondria, encoding structural OXPHOS proteins or mitochondrial ribosomal proteins (Figure S1b). Surprisingly, the 30 kbp region in chromosome 2 is transcribed at similar levels as the one found in the mitochondrial genome (Figure S1b). However, we presume that the transcripts of the nuclear version of *COX3* are not edited and the protein, if synthesized, is probably not able to be imported into mitochondria. The gene coding for COX2 is absent in the mitochondrial genome and has migrated to the nucleus in *Glycine max* and other leguminous plants. In this case, Gm*COX2* seems to be fully edited and also acquired a pre-sequence for import into mitochondria, thus allowing the replacement of the mitochondrial *COX2* copy [65].

We also identified eight genes that encode three accessory COX subunits with sequence homology to yeast and human proteins (COX5b, COX6a and COX6b). It is noteworthy that the number of isoforms of COX5b and COX6b is higher in Arabidopsis than in the other organisms (Table 1). For COX5b, one of the genes, *COX5b-3*, encodes a smaller isoform due to a deletion in the N-terminal region. No evidence for the existence of the corresponding protein is available. For COX6b, one long (COX6b-1; 191 amino acids) and two short (COX6b-2 and COX6b-3; 78 amino acids) forms are present. A fourth isoform (COX6b-4) has an intermediate length and has not been identified experimentally. It is remarkable that homologs for several of the subunits conserved in mammals and yeast were not identified in Arabidopsis (Table 1). Several of these are small proteins of about 100 amino acids or less, which may hinder their identification if their sequence diverged. However, putative homologs of these subunits were not identified in proteomic studies of mitochondrial complexes, suggesting that plant COX differs in this respect from its yeast and human counterparts [59,66]. Additionally, proteomic studies identified a set of subunits that seem to be plant-specific. COX5c was identified in early studies of plant COX purification. Putative additional subunits, named COX-X, were also identified [59,60]. Several of them co-migrate with other COX subunits in Blue Native Polyacrylamide Gel Electrophoresis (BN-PAGE), suggesting that they are structural COX subunits (Figure S2). In summary, plant COX retains the catalytic-core subunits, which are universally conserved in eukaryotes and prokaryotes, and a few additional structural subunits, while several subunits are not conserved and seem to be plant-specific. It is possible that this is due to extensive sequence divergence, but also that the evolutionary acquisition of new accessory subunits was different in plants than in fungi and mammals. This implies that some aspects of the COX assembly process may also differ in plants. It is surprising that two of the conserved subunits, COX6a and COX6b, are non-essential subunits, while many yeast and human essential subunits do not seem to be present in plants. COX6a and COX6b have been implicated in the formation of COX oligomers (either dimers or supercomplexes), and their conservation suggests an ancestral role and the importance of oligomerization for COX function. Related to this, putative homologs of Rcf1 and Rcf2, required for supercomplex formation in yeast, are also present in plants [67]. In addition, a putative homolog of the COX assembly factor COX16 is also present in Arabidopsis. Recently, Cox16 was found associated to Cox1 assembly intermediates in yeast, but also as a substoichiometric subunit in mature COX and in supercomplexes, suggesting that it may also be a non-essential subunit that regulates COX properties or oligomerization [68].

Several putative COX assembly factors were also identified by sequence homology in Arabidopsis. The five factors already present in prokaryotes required for co-factor assembly (COX10, COX15, COX11, SCO and SURF1) are present in *Arabidopsis* (Table 1 and Table S1). Single genes encode the first three proteins, and those encoding COX10 and COX11 have been characterized, confirming their function as COX assembly factors [17,69]. SCO proteins are encoded by two genes, named *HCC1* and *HCC2*. These genes were also characterized, and only HCC1 seems to be required for COX assembly [18,19,20]. HCC2 is present in angiosperms and gymnosperms and has lost the conserved cysteines and histidines required for copper binding. Current evidence suggests that HCC2 function is related to copper and stress responses in plants [18]. For SURF1, also two genes, that seem to be the consequence of a recent duplication within the *Brassicaceae*, can be recognized in Arabidopsis.

Putative homologs of other eukaryotic proteins known or supposed to be involved in copper trafficking and insertion, like COX17, COX19, COX23, COA6, CMC1, CMC2, and PET191, were also identified. Arabidopsis genes encoding COX17 and COX19 (two genes for each protein) have been characterized and are able to complement the corresponding yeast *null* mutants, suggesting that the proteins are indeed COX assembly factors [70,71,72]. In addition, COX17 was involved in the regulation of plant stress responses [72,73]. Homologs of two proteins required for heme A synthesis, Yah1 and Arh1 in yeast, are also present in *Arabidopsis*. These proteins are mitochondrial ferredoxin and ferredoxin reductase, respectively, and are required for electron transfer reactions related to the conversion of heme O to heme A. It is not known if the Arabidopsis proteins are required for heme A synthesis. It was reported that they may participate in biotin synthesis instead [74], and it is likely that they have general roles in electron transfer reactions in the mitochondrial matrix.

Interestingly, homologs of COX18 and COX20, specifically required for the insertion of core COX subunits into the IMM, were not identified. Instead, two OXA1 insertase homologs are present in Arabidopsis [75]. One or both of these proteins may be functional in COX subunit insertion. As mentioned above, OXA1 participates in this process in yeast but apparently not in humans [76], but COX18 and COX20 are required in both organisms. Arabidopsis also contains two additional mitochondrial proteins, named OXA2, with lower sequence homology to yeast Oxa1 and that may participate in the assembly process [77]. Homologs of the Imp1/2 IMM peptidase are also present in plants. It is not known if they are particularly involved in COX2 biogenesis, since they probably have a general function in IMM protein translocation. As a general rule, assembly factors involved in the biogenesis of the redox-active centers are present in plants, while most COX-specific factors involved in other aspects of COX biogenesis seem to be lacking. Considering the different subunit composition of plant COX, probably different factors, yet to be discovered, are involved in this process. 

Among the proteins required for COX biogenesis in yeast, several of them are RNA binding and/or processing proteins involved in RNA maturation or translational regulation within mitochondria. Proteins with sequence homology to some of these proteins are found in *Arabidopsis*, but it is not clear if they are involved in COX biogenesis. Only proteins that present a *p*-value < 10^−8^ about their sequence identity with RNA processing proteins previously identified in yeast or mammals were included in Table 1. Plant mitochondria have a complex RNA metabolism that involves hundreds of proteins. Unlike mammals and yeast, flowering plants must convert hundreds of cytidines to uridines in their chloroplast and mitochondrial transcripts through a post-transcriptional process known as RNA editing. To successfully carry out this process, plants have editosome complexes comprised of pentatricopeptide repeat (PPR) proteins [8], and non-PPR editing factors, including RIPs/MORFs, ORRMs, OZs and protoporphrinogen IX oxidase 1 (PPO1) (recently reviewed in [9]). Several proteins have been experimentally demonstrated as responsible for the editing and maturation of transcripts encoding mitochondrial COX components, and these are listed in Table S1 and described in Table 2 in detail.

We analyzed the possible arrangement of COX subunits and assembly factors into protein complexes or supercomplexes using the server Complexome (https://complexomemap.de/at_mito_leaves/ [59]). Forty-three proteins were detected in the database, built with proteins isolated from Arabidopsis leaves [59]. As expected, structural subunits, including several COX-X subunits, were grouped together in 2 bands migrating at about 210 and 300 kDa. The most abundant band (210 kDa) does not contain COX6b-1, the larger form of COX6b, which is present in the 300 kDa complex (Figure S2). The Rcf1 homolog ATL48 also migrates with other COX subunits, suggesting that it may be part of plant COX. In yeast, it was proposed that Rcf1 is not a stoichiometric COX subunit and that it rather acts as a late-maturation factor. The human homolog, HIGD1A, forms a complex with early COX1 assembly intermediates. A group of COX subunits also appears at 700 and 1700 kDa, probably reflecting previously described III_2_ + IV and III_2_ + IV + I supercomplexes [99]. COX11, HCC1, HCC2 and SURF1-1, all migrated similarly in a region slightly below 100 kDa. The MW calculated for these proteins is between 30 and 40 kDa, which suggests that they are probably dimers or trimers or that they interact with other proteins (Figure S2). The formation of dimers was suggested for SCO proteins from humans. All of these proteins are also present in regions of slower migration, perhaps reflecting their incorporation into assembly complexes. COX15 forms oligomers of about 120 kDa, probably also dimers or trimers. Oligomerization of Cox15 in yeast was suggested to be important for its function. Homologs of Pet117, involved in Cox15 oligomerization, were not found in Arabidopsis. Unfortunately, the migration patterns of COX1 and COX3 are not provided in the Complexome database, not allowing the identification of putative assembly intermediates containing these proteins.

## 4. Factors Regulating COX Assembly and Activity

### 4.1. Regulation of Gene Expression

#### 4.1.1. Tissue- and Organ-Specific Expression

**Figure 1:**
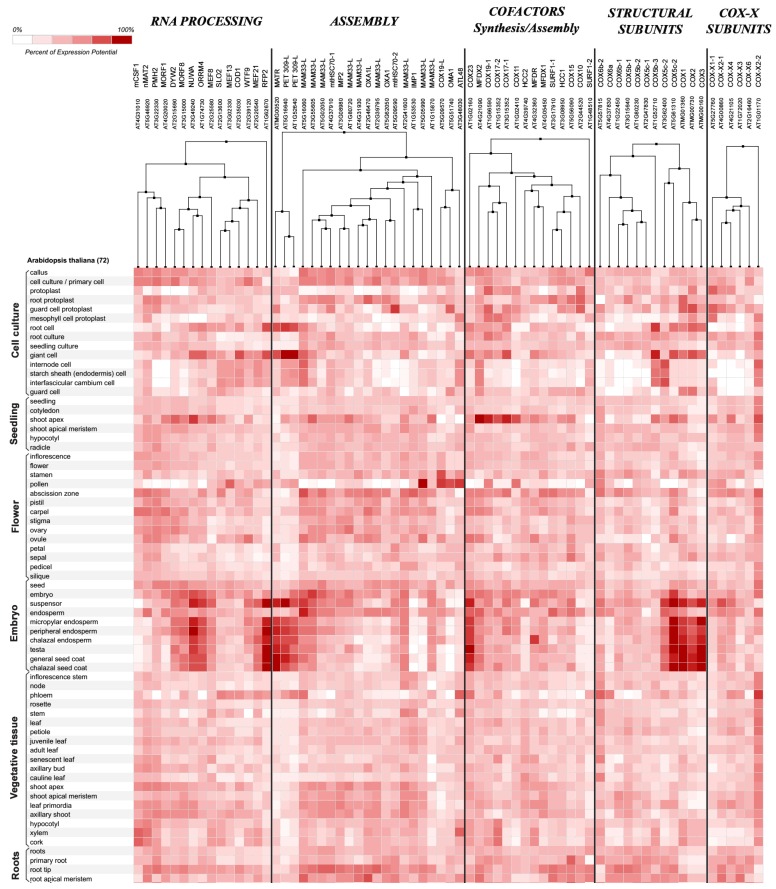
Hierarchical clustering of expression data across different tissues, cell types and developmental stages. Meta-analysis of the expression of genes encoding 68 COX-related proteins according to tissue- and cell-type, and in different developmental stages. Candidate proteins were classified into five different categories according to their putative or demonstrated role in COX biogenesis. Hierarchical clustering was performed within each category. Transcriptional data were collected and analyzed using publicly available microarray data included in the Genevestigator database (https://genevestigator.com/gv/doc/intro_plant.jsp, [100]). The expression level is represented as percent of maximal expression in the dataset analyzed.

**Figure 2:**
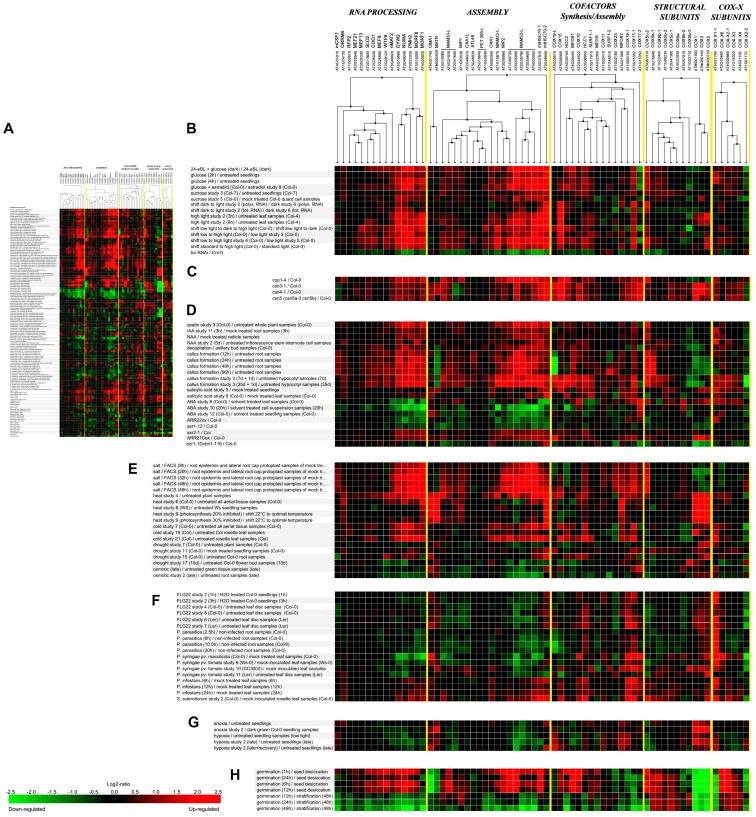
Meta-analysis of transcriptional data for genes encoding COX-related proteins in response to different perturbations or in several mutant backgrounds. (**A**) Complete hierarchical clustering of the expression data. A larger image is available in Figure S3. (**B**–**H**) Detail of specific parts of the clustering for transcriptional responses to nutrients and light (**B**); in mutants in members of the COP9 signalosome and the *cop1-4* mutant (**C**); in response to hormones (**D**); abiotic stress (**E**); biotic stress (**F**); during oxygen deprivation (**G**); and during germination (**H**). Expression level is represented as log2-ratio of differential expression, in red for up-regulation and in green for down-regulation.

To analyze the transcriptional regulation of the expression of genes encoding COX-related proteins, we performed a meta-analysis by exploring publicly available microarray data included in the Genevestigator database (https://genevestigator.com/gv/doc/intro_plant.jsp [100]). We obtained information about the expression profile of 67 genes according to organ/tissue, plant developmental stage, (Figure 1) and responses to several perturbations (Figure 2 and Figure S3). Hierarchical clustering of the expression data across different tissues and cell types obtained from the “Anatomy” and “Development” tools showed that several genes for COX structural components or editing and assembly factors are preferentially expressed in roots and endoreduplicative giant cells, in the shoot apical meristem, and at different stages during embryogenesis (Figure 1). This agrees with previous experimental analysis of the expression patterns of some of these genes [17,18,19,72,101,102,103,104,105,106,107], suggesting that they are expressed in tissues with high energy demands. In agreement with the central role of mitochondria in cellular metabolism and as energy suppliers during developmental processes and cellular differentiation, COX biogenesis seems to be regulated at different stages of development, and according to organ and tissue type.

#### 4.1.2. Expression of Different Isoforms

In mammals, 6 of the 11 nuclear-encoded subunits possess differential tissue- or environmental condition-specific isoforms [7]. This may result in COX isoforms with different activity or regulatory properties, different affinity for oxygen, or different interactions with other OXPHOS components [6,7]. In Arabidopsis, some genes coding for different COX isoforms that theoretically fulfill the same function show specialized expression characteristics. As examples, *COX5b-1* and *COX5b-2* are preferentially expressed in meristematic regions or in vegetative tissues and are differentially regulated by external stimuli [104,108,109,110]. This is related to the presence of different *cis* regulatory elements in their promoter regions [111]. Interestingly, the expression patterns and responses of *CYTC-1* and *CYTC-2*, both genes encoding the electron carrier CYT*c*, which is required for complex IV stability [97], are similar to those of *COX5b-2* and *COX5b-1*, respectively [105,106]. In addition, they contain similar responsive elements in their promoters, suggesting the existence of co-evolution in their expression mechanisms. A model where one of the genes maintained its ancient expression characteristics while the other one incorporated novel elements that allowed a progressive specialization was proposed [109]. Whether the different isoforms confer different properties to plant COX is not known.

As a possible example of functionalization, the At*COX19-1* gene produces two transcripts by alternative splicing that originate proteins with different N-terminal ends. The smaller AtCOX19-1 isoform is the only one able to restore growth of the yeast *cox19* null mutant [101]. A more extreme case of divergence is represented by genes encoding plant SCO proteins. AtHCC1 is the Arabidopsis homolog of yeast Sco1, involved in copper delivery to COX, while AtHCC2 functions in copper sensing and stress responses, but does not seem to be required for COX assembly [18,19,20].

#### 4.1.3. Response to Nutrients

We also performed a meta-analysis for transcriptional data related with “Perturbations” available in Genevestigator [100]. As a criterion of selection for posterior analysis, we included all experiments in which at least 30% of genes included in the analysis were differentially expressed with a fold change higher than 2. We applied a clustering analysis within individual gene categories and the images were fused to improve visualization of the results (Figure 2; see Figure S3 for a magnification of Figure 2A).

Differential expression of mitochondrial genes (*COX1*, *COX2*, *COX3* and *MATR*), relative to nuclear genes, was observed, as reported previously [112]. Higher expression of nuclear genes, mainly those related to editing and assembly factors, was observed in plants treated with glucose or shifted to high-light conditions. An opposite behavior was observed in plants in which the target of rapamycin (TOR) kinase, a regulator of plant growth in response to nutrients [113], was silenced (Figure 2B). Strengthening the connection between light, development and mitochondria, a marked increase in expression is observed in mutants of the members of the COP9 signalosome (*csn3-1*, *csn4-1*, and *csn5*), and in the *cop1-4* mutant [114], with altered photomorphogenesis (Figure 2C). It was previously observed that transcript levels for *COX5b*, *COX5c*, *COX6a* and *COX6b* genes are regulated by light, metabolizable sugars and nitrogen sources [102,103,104,107,108,109,110,115,116], and that carbohydrates produce an increase in transcript levels for nuclear genes but not for the mitochondrial ones [102,112]. It was proposed that carbon and nitrogen sources act in concert to regulate the expression of different components of the respiratory pathway [117].

We evaluated the presence of common regulatory elements in the promoter regions of nuclear COX-related genes. To achieve this, the sequences corresponding to the first 500 bp upstream of the transcriptional start site of each gene were obtained and loaded in the MEME server [118]. Three elements were statistically over-represented: wwTGGGCY (*p* < 10^−29^), rAAgAArA (*p* < 10^−15^), and TTTTkTTT (*p* < 10^−2^) (Figure S4). The last two motifs do not match with any previously recognized regulatory element present in the TOMTOM database (http://meme-suite.org/tools/tomtom; [118]). The element wwTGGGCY matches with the previously reported Site II element (TGGGCY) [117,119]. Site II elements are present in the promoter regions of genes encoding COX components [105,106,107,108,117,119]. This regulatory motif has been reported as relevant in regulation of gene expression in meristematic regions [105,119], in the response to light and carbohydrates [117,119], in the coordination of gene expression for the biogenesis of the OXPHOS machinery [117], in the connection between the expression of mitochondrial proteins and the circadian clock [120], as well as in coordinating the expression between chloroplast, peroxisome and mitochondrial components [1].

#### 4.1.4. Response to Hormones

We described evidence that COX activity is connected to plant growth and development, and there are several examples that alterations in COX activity seriously impact on plant fitness (Table 2). This is somewhat unsurprising, considering that mitochondrial OXPHOS is probably required for obtaining the energy necessary for these processes. Interestingly, the opposite also seems to happen, since a regulation of plant growth programs over COX exist. Expression of nuclear genes coding for COX proteins is particularly connected to hormonal control of plant growth. Plant hormones are connected to mitochondrial activity through abscisic acid (ABA), auxins, cytokinins (CKs), jasmonic acid (JA), and salicylic acid (SA), regulators of the expression of nuclear genes encoding mitochondrial components [121]. In this sense, *COX6b-1* is induced by gibberelins (GA_3_) [107], ABA increases the expression of *COX5b-1* and *CYTC-2,* and *COX6b-3* is induced by CKs [105,106,107,111]. A recent study demonstrated that CKs can differentially activate the alternative respiratory pathway in roots subjected to oxidative stress, decreasing reactive oxygen species (ROS) production and drought-induced oxidative stress, and thus promoting root growth [122]. Furthermore, the expression of the metallochaperones AtCOX17 and AtCOX19 is induced by SA, a hormone connected to biotic stress [72,73,74,75,76,77,99,100,101]. Hormones also seem to have impact on the posttranslational regulation of COX assembly factors. AtCOX17 and the hypoxia regulated protein AtATL8 are differentially phosphorylated in response to auxins [123], while the same effect was seen in AtMATR after treatment with brassinosteroids (BRs) [124].

The analysis presented in Figure 2D allows the discrimination of different responses of COX-related genes to hormones or inhibitors of hormonal pathways. There is an increased expression of several RNA-processing and assembly proteins in aerial plant tissue treated by CK (zeatin), in roots and inflorescence stem internode tissues exposed to the auxins IAA (indole-3-acetic acid) and NAA (1-naphthaleneacetic acid), and during the first 96 h in callus-inducing medium. During the last stages of callus formation, there is an enhancement of the expression of nuclear genes for structural proteins, while mitochondrial genes are down-regulated. Among the biotic stress-related hormones, SA causes a general weak induction that is more pronounced for genes encoding co-factor synthesis or assembly proteins. A clear opposite behavior is observed after ABA treatments, where all genes seem to be down-regulated.

When mutants in hormonal synthesis and signaling pathways are evaluated, opposite expression is observed for RNA processing and assembly proteins in plants with altered response to auxins. While in *axr2-1* mutants, where auxin responses are impaired [125], they are up-regulated, in *axr1-12* auxin-resistant plants their expression is decreased. The same behavior is observed in plants overexpressing the *A. thaliana* response regulators (ARRs) ARR21C or ARR22, being the expression down-regulated in ARR22ox plants, that exhibit a dwarf phenotype previously associated with CK deficiency [126]. Gene expression is also decreased in a *bzr1-1D* mutant background [127], in which the brassinosteroid (BR) signaling pathway and several aspects of plant growth and development are impaired (Figure 2D).

Mitochondrial dysfunction due to alterations in mitochondrial respiratory proteins has been associated to a decrease in auxin responses [128,129]. Future experiments will be required to establish the molecular network connecting hormones and COX biogenesis/function.

#### 4.1.5. Stress Conditions

Mitochondria fulfill a central role during perception, signaling and orchestrated responses to biotic and abiotic stress in plants [130,131,132,133]. In relation to this, transcript levels for At*COX17* and At*COX19* are increased by exposition to toxic concentrations of Cu^2+^, Zn^2+^ and Fe^2+^, to compounds that produce ROS, and after wounding and infection with the pathogen *Pseudomonas syringae* [72,101]. The silencing of At*COX17* genes originates a decreased response of several stress genetic markers, including those that comprise the mitochondrial retrograde response and are normally induced by mitochondrial dysfunctions [73]. It was proposed that AtCOX17, in addition to its function as a copper metallochaperone for COX assembly, acts as a component of signaling pathways that link plant stress to gene expression responses [73]. As another example, *AtCOX5b-2* is induced in *cyp79B2/cyp79B3* mutants, deficient in aliphatic glucosinolates, important secondary metabolites in the defense against insects and pathogens [134].

Analysis of transcriptional data in response to different stress situations (Figure 2E) shows a coordinate increase in the expression of several RNA processing and assembly factors with decreased expression of mitochondrial genes in root cells exposed to salt. The opposite occurs during heat stress, where COX catalytic-core genes are highly induced, while no changes are observed during cold treatments. Drought and osmotic stress do not generate a marked behavior.

Considering the responses to biotic stress, a decrease in the expression of several nuclear genes is observed when plants are exposed to elicitors (Flg22), bacterial pathogens (*P. syringae*), or the oomycete *P. parasitica*. A contrasting behavior is observed for mitochondrial genes and those nuclear genes coding for the metallochaperones HCC1, COX19, COX17-1, and COX17-2, among a few others, in agreement with previous results [72,101] (Figure 2F). A general increase in expression is observed during infection with necrotrophic pathogens like *Phytophthora infestans* or *Sclerotinia sclerotiorum* (Figure 2F). One possibility is that COX assembly or structural proteins accumulate under certain stress conditions to actively replace damaged or inactive COX subunits to avoid or diminish ROS generation due to a dysfunctional mitochondrial cyanide-sensitive respiration.

### 4.2. ROS- and Redox-Regulated Mechanisms Involved in COX Biogenesis 

#### 4.2.1. Regulation by Oxygen Availability

Cellular respiration consumes oxygen as terminal acceptor of electrons from reduced equivalents produced by the cell during metabolism. In mammals and yeast, different COX isoforms, with different affinity for oxygen, presumably exist. The expression of subunits Cox5a and Cox5b from yeast, and COX4-1 and COX4-2 from mammals, is differentially regulated by oxygen tension, being the isoform expressed under hypoxia responsible for increasing COX catalytic efficiency. This adapts the respiratory electron transfer rate to environmental requirements [135]. As another example, expression of the human *COX10* gene is regulated by the microRNA miR-210, which is induced under hypoxia to reduce COX10 levels and thus regulates the rate of oxygen consumption and mitochondrial metabolism in conditions of low oxygen availability [136]. In plants, transcript levels of genes encoding rice COX5b and COX5c and sunflower COX5c are severely reduced under hypoxia and recovered to initial levels when plants are returned to normal aerobic conditions [112,137]. Transcriptomic data indicate that nuclear genes are down-regulated under anoxia and hypoxia in Arabidopsis, whereas mitochondrial genes appear to be up-regulated (Figure 2G). This suggests that COX biogenesis may also be under the control of oxygen availability in plants.

#### 4.2.2. Regulation of Import and Oxidative Folding

Mitochondria are particularly important in regulating ROS-mediated processes [138]. During respiration, ROS are inevitably produced, mainly in Complexes I, II and III [139]. Paradoxically, COX, that should be one the major oxygen consumers in the cell, does not generate ROS, but its biogenesis involves several ROS and redox regulated steps [135]. While cells have evolved strategies to avoid the generation of pro-oxidant compounds during COX biogenesis, redox biology and ROS exert a control during the assembly and copper delivery to the complex. Mitochondria are rich in thiol-containing proteins that, together with the couple reduced/oxidized glutathione (GSH/GSSG), are specifically compartmentalized in the matrix and the IMS [140,141,142]. Many IMS proteins that are synthesized in the cytosol are imported through a conserved redox-regulated disulfide relay system established by the receptor MIA40 and the sulfhydryl oxidase ERV1 [143]. Some of the imported proteins, and MIA40 itself, share conserved CX_3_C or CX_9_C motifs. Thirteen of the twin CX_9_C family members are involved in COX biogenesis in yeast and are conserved in animals and plants [144]. In mammalian cells, the kinetics of import through the MIA40/ERV1 system depend on the glutathione pool, thus providing a connection between the redox state of the IMS and the amount of imported CX_9_C proteins [145]. In plants, while AtERV1 was found to be critical for mitochondrial biogenesis, AtMIA40 seems to be dispensable for import to the IMS [143,146] and recent evidence suggests that it only improves substrate specificity [147]. A second connection between the MIA40-ERV1 import pathway and respiration is established by CYT*c*, the final electron acceptor of this pathway that transfers them to COX [148,149]. Thus, the cellular redox state, and more precisely that of the IMS, defines the amount and the proper oxidative folding of CX_9_C proteins imported by the MIA40/ERV1 system, which is connected to the respiratory chain and, at the end, to the energetic status of the cell. 

#### 4.2.3. Regulation of Copper Metalation

Copper insertion into COX1 and COX2 involves several redox-dependent conformational changes of metallochaperones from the IMS [2,150,151]. COX17 forms disulfide bridges and changes its oligomerization state depending on the redox state, thus adopting different conformations for copper transfer to COX [34,35]. Besides Cox17, other proteins with twin CX_9_C motifs reside in the IMS and were demonstrated as essential for copper uptake and COX assembly in yeast and humans (Table 1): CMC1, CMC2 [27,152], COX19 [153,154], COX23 [155], PET191 [156], and COA6 [157,54]. CX_9_C proteins could also play a role in copper transfer towards COX17 by modulating the local IMS redox environment [135,158]. In addition, the redox state of human SCO1 modulates the copper content of cells, and COX19, that partitions between the cytosol and the IMS in a copper-dependent manner, was postulated as a transducer of the signal that connects SCO1 with copper transporters of the plasma membrane [159].

COX assembly factors responsible for metalation of the catalytic centers have their counterparts in plants. Two Arabidopsis genes, At*COX17-1* and At*COX17-2*, encode functional plant homologs able to complement the respiratory deficiency of a yeast *cox17* null mutant [70,71,72]. While plants in which both *AtCOX17* genes are silenced show severe phenotypic changes, presumably due to decreased COX activity, the silencing of either gene does not alter plant growth [73]. However, these plants show altered ROS levels and a reduced response to stress conditions. Arabidopsis also encodes two proteins with homology to yeast Sco1, named HCC1 and HCC2 (*Homolog to yeast Copper Chaperone*), but HCC2 lacks the canonical copper-binding motif described in Sco1 [18,19]. Several pieces of evidence point to HCC1 as a COX assembly factor in plants: (i) HCC1 is able to complement a yeast *sco1* mutant [19]; and (ii) knockout of *HCC1* produces embryo arrest, while heterozygous mutants show a decrease in COX activity [18,19,20] (Table 2). In addition, knockout plants in *HCC2* show normal COX activity levels and no obvious phenotype under normal growth conditions [18,19,20]. Changes in the expression of HCC1 and HCC2 also cause altered redox homeostasis and responses to copper and stress conditions [18,19,20]. *Arabidopsis* also contains a COX11 homolog that seems to be essential for normal COX activity. Altered levels of this protein affect pollen germination, development, growth, and copper homeostasis [69] (Table 2). Finally, two different genes, At*COX19-1* and At*COX19-2*, encode putative homologs of yeast Cox19 [101]. Altered levels of AtCOX19 impact on plant phenotypic responses to changes in copper and iron levels in the growth medium and in the expression of metal-responsive genes. 

#### 4.2.4. Heme A Synthesis and COX1 mRNA Translation

Within the process of Complex IV formation, Cox1 hemylation is essential and, since free heme is toxic to the cell, its synthesis and insertion must be precisely coordinated. Studies in yeast established that Cox1 is translationally regulated by heme B availability [4], while heme A biosynthesis is regulated by the redox-dependent oligomerization of Cox10 (heme O synthase) [21,51]. After Cox1 synthesis, Mss51, a *Cox1* mRNA-specific processing factor and translational activator, forms a Cox1 preassembly complex that is only disrupted when the prosthetic groups are inserted into Cox1. Formation of this complex limits the availability of Mss51 for *Cox1* translational activation, thus linking Cox1 synthesis to COX assembly. It was recently demonstrated that Mss51 is activated by heme B and that the redox environment modulates the affinity of Mss51 for heme, thus compromising Mss51 function in *Cox1* mRNA translation [11]. In addition, Pet54 is required for the activation of *Cox1* mRNA translation by Mss51, probably influencing its hemylation or conformational state [160]. Coordination of COX biogenesis with heme availability and the redox state are probably important to avoid the accumulation of pro-oxidative reactive assembly intermediates and to minimize the potential damaging effects of ROS. Nothing is known about the regulation of the synthesis of COX1 and other subunits encoded in mitochondria in plants.

### 4.3. Regulation by Phosphorylation

Phosphoproteomic studies identified several phosphorylated proteins in purified mitochondria from Arabidopsis cell suspensions [161]. Recent studies in mammalian and yeast mitochondria demonstrate that reversible phosphorylation of proteins on serine, threonine and tyrosine residues is an important biological regulatory mechanism across a broad range of important mitochondrial processes. In mammals, there is solid evidence that COX activity is regulated by phosphorylation and, to date, between 14 and 18 in vivo phosphorylation sites were identified by mass spectrometry (MS) in several Complex IV proteins [162,163]. In some cases, connections of the phosphorylation state of COX components with respiratory energy demands, human diseases, and cell destiny mediated by apoptosis were established [162,163]. In plants, phosphorylation of COX components is less studied and there is discrete evidence that this PTMS specifically impacts on COX proteins in plants exposed to nutritional deficiencies or exposed to hormones (Table S1). AtCOX5b-1, AtCOX6a and several PPR proteins are differentially phosphorylated in a *atm/atr* serine/threonine protein kinase double mutant when plant rosettes are exposed to ionizing DNA-damaging radiation [164]. Furthermore, AtCOX5b-1, AtCOX6a, several PPR proteins and members of the glycoprotein family homologous to MAM33 from yeast were identified in a proteome-wide profiling of phosphopeptides in nine-day-old Arabidopsis seedlings [94]. These proteins were proposed as part of a phosphor-regulatory network involved in biological processes like central metabolism and cell signaling, regulating plant growth and development [161,94]. Several MAM33-like proteins, AtHCC1 and AtSURF1-2 were identified as differentially phosphorylated when nitrogen sources are supplied to growing Arabidopsis seedlings in the form of nitrate or ammonium [165]. As mentioned, AtCOX17 and AtATL8 are differentially phosphorylated in the presence of auxin when this hormone is tested in a lateral root-inducing system [123], and AtMATR2 is part of a group of proteins that are rapidly modified by phosphorylation in response to BRs [124]. To conclude that these modifications have real impact on the regulation of COX activity and are connected to mitochondrial energy generation by respiration or to additional functions will require an in-depth future analysis. 

## 5. Mutant Plants with Altered Cox Activity

In this section, we present a survey of plant mutants related to COX assembly or mutants in which COX activity is, either directly or indirectly, altered (Table 2). This may be useful to understand the role of COX in plant physiology and development.

### 5.1. Mutants in COX Assembly Factors

The silencing of *AtCOX17-1* in a *cox17-2* knock-out background generates plants with small rosettes and severely delayed development [73]. Silencing of *COX17-1* or *COX17-2* independently does not have any phenotypic consequence and does not affect COX activity, but causes a decrease in the response of plants to stress [73]. COX11 knock-down plants show reduced COX activity [69]. In addition, they exhibit a short root phenotype and reduced pollen germination [69]. HCC1 is essential for plant development [18,19]. *AtHCC1/athcc1* heterozygous mutant plants produce 25% defective seeds with embryos that stop their development at the heart stage [18,19]. Rescue of *hcc1* knock-out embryo lethality by complementation with the *HCC1* gene under the control of the embryo-specific *ABSCISIC ACID INSENSITIVE 3* promoter caused growth arrest at early plant developmental stages, supporting the role of HCC1 for plant growth and development [20]. Heterozygous *AtHCC1*/*athcc1* mutants show a ca. 50% decrease in COX activity and no phenotypic alterations. Homozygous loss-of-function mutants in *AtHHC2* do not affect plant development or COX activity.

Null mutations in *AtCOX10* also lead to embryonic lethality [17]. Heterozygous *AtCOX10*/*atcox10* plants show reduced COX activity and normal levels of total respiration at the expense of an increase in alternative respiration. In addition, AtCOX10 seems to be implicated in the progression of senescence, since heterozygous mutants show an increased rate of chlorophyll loss and photosynthesis decay during both natural and dark-induced senescence [17].

### 5.2. Mutants in RNA Processing Factors Influencing Mitochondrial COX-Coding Genes

There is a group of mutant plants where the mutation affects nuclear-coding genes that are necessary for correct mitochondrial RNA processing, including protection of 5′ and 3′ termini, splicing and editing, that constitute a relevant tool to understand the effects of defective COX over plant growth [98]. RNA editing in land plant organelles alters the coding content of transcripts through site-specific exchanges of cytidines into uridines and vice versa. In model plant species such as rice or *Arabidopsis*, there are about 500 editing sites, indicating the importance of this mechanism [166,167]. One of the most important plant-specific protein family involved in RNA editing is the PPR protein family. PPR proteins are sequence-specific RNA binding proteins that identify C residues for editing. All the reported PPR proteins required for RNA editing are members of E or DYW subclasses of the PPR family [84,168,169].

PpPPR_78, identified and characterized by Uchida et al. [84], is a protein involved in *COX1* editing in *Physcomitrella patens.* Disruption of the *PpPPR_78* gene results in editing defects in *rps_14* (*rps14-C137*) and *cox1* (*cox1-C755*), with no changes in other editing sites [85]. At the phenotypic level, mutant plants display slight growth retardation. This suggests that the editing events are not crucial for RPS-14 and COX1 function. PpPPR_77, another member of the PPR protein family from *Physcomitrella patens* was described by Othani et al. [86] as a mitochondria-localized protein involved in RNA editing. PpPPR_77 mutants show editing defects in *cox2* (*cox2-C370*) and *cox3* (*cox3-C733*) sites, but not in other editing sites. The mutant has severe growth retardation.

*Arabidopsis* Cytochrome *c* Oxidase Deficient 1 (*COD1*) encodes a mitochondria-localized PLS-subfamily PPR protein involved in the editing of two distant sites in the *COX2* transcript [80]. Lack of COD1 generates embryo lethality, since no homozygous mutant plants could be identified in the progeny. Immature homozygous mutant seeds could be germinated using a high-sugar medium supplemented with nutrients and co-factors. However, their subsequent growth was retarded, and plants showed a proliferation of short leaves with limited stems and a delay in root development. Occasionally, some plants were able to produce flowers which did not produce viable pollen. COX activity could not be detected in mutant lines and no signal of native Complex IV was observed using anti-COX2 antibodies. MEF13 is a E domain PPR protein required for editing at eight sites in *Arabidopsis thaliana*, including *COX3*-*314* [81]. *mef13-1* mutants exhibit delayed growth at the vegetative phase but no defects were detected at later stages. 

*DEK10* encodes a E-subgroup PPR protein from maize that is specifically involved in the C to U editing at *nad3*-*61*, *nad3*-*62* and *cox2*-*550* [83]. Defects in these editing events reduces the function of Complex I and Complex IV in the electron transport chain and affect mitochondrial respiration and embryo, endosperm and seedling development. Homozygous *dek10* mutant kernels are small and their weight is 73% reduced compared to wild-type. Vegetative growth is also severely delayed in the mutants. Lack of DEK10 affects COX2 protein accumulation, while Complex I and Complex IV activities are reduced compared to wild-type.

In addition to PPR proteins, other protein families were also identified as RNA editing factors in mitochondria. One of these families is called ORRM (Organelle RNA Recognition Motif-containing protein) and plays a crucial role in mitochondrial RNA editing in *Arabidopsis thaliana* [9,82]. The absence of ORRM4 expression causes defective mitochondrial editing at 264 sites in 31 mitochondrial transcripts, among them *COX2* and *COX3*. *Orrm4* mutant plants show slow growth and the average flowering time is delayed three days in comparison with wild-type plants.

MORF family proteins (multiple organellar RNA editing factor) are required for RNA editing in plant mitochondria as additional components of the RNA editing machinery [79]. The etilmetanosulfonato (EMS)-mutated Arabidopsis *morf1* mutant shows reduced RNA editing at more than 40 mitochondrial sites, without defects in other RNA processing steps or RNA stability. *COX3-257* is one of the sites affected by *MORF1* mutation, with a 50% decrease in editing events of *COX3* transcripts. Homozygous mutant plants with T-DNA insertion in the *MORF1* gene are not viable, due to the abortion of seed development. This finding suggests that this protein is essential and that the EMS mutant is hypomorphic and that residual MORF1 activity is sufficient for plant viability. 

Plant mitochondrial gene expression involves the splicing of introns from the coding regions. The mitochondrial introns of angiosperms are classified as group II-type. Particularly in Arabidopsis, these include 23 introns found within Complex I, CYT*c* biogenesis factors, Complex IV and ribosomal protein genes. This process relies on many different RNA-binding proteins or co-factors. Two members of the maturase and RNA helicase families, nMAT2 and PMH2, participate in the splicing of several transcripts in *Arabidopsis* mitochondria, including *COX2* [89]. *mat2/pmh2* double mutants show a decrease of mRNA levels of multiple mitochondrial genes, and exhibit growth delay and alterations in vegetative and reproductive development. No change in total respiration was detected. However, inhibition of Complex IV by KCN appeared to have a reduced effect on the respiratory activity of *mat2/pmh2* plants.

Besides their role in editing, the PPR protein family is also involved in other steps of RNA metabolism in plant organelles. Particularly, PpPPR_43 has been reported as a splicing regulator of *COX1* mRNA in *Physcomitrella patens* [90]. The *PpPPR_43* disruptants show normal editing, but mature *COX1* transcripts levels are substantially reduced. Instead of the mature *COX1* transcript, unexpectedly long transcripts were detected. A defect in PpPPR_43 strongly inhibits the splicing of *COX1* intron 3, without affecting the splicing of introns 1, 2 and 4. Mutant plants show severe growth retardation.

Most 5′ termini of plant mitochondrial transcripts are generated post-transcriptionally [170]. RFP2 is a PPR protein involved in the formation of *COX3* 5′ transcript ends. It has been reported that *COX3* mRNA 5′-end formation is affected in *rfp2* mutants, resulting in a substantial reduction of mature *COX3* transcripts. However, the impaired 5′ processing does not interfere with protein accumulation.

The plant organellar RNA recognition (PORR) domain was recognized to be an RNA binding domain, and most of the members of this family are involved in mRNA splicing in chloroplasts and mitochondria. WTF9 is involved in the splicing of group II introns in mitochondria and is required for the CYT*c* maturation pathway [87]. Mutant plants lacking WTF9 show severely stunted growth. They survive to flowering but the flowers are small with very small anthers and small amounts of pollen. This results in very small siliques with a few aborted seeds. The levels of CYT*c*, cytochrome *c*_1_, and the COX2 subunit are severely reduced in mutant mitochondria, which is accompanied by a dramatic increase of the alternative oxidase, which is commonly induced when the respiratory chain is compromised.

Not all Mitochondrial Editing Factors (MEFs) are essential for plant growth. This is the case for MEF21 [171] or MEF26 [172], two RNA processing factors belonging to the E- or DYW-groups of PPR mitochondrial proteins, respectively. The *cox3-257* site targeted by MEF21 changes a Ser to a conserved Phe residue at amino acid 86 in COX3 [171]. MEF26 is involved in editing of *cox3-311* and *nad4-166* sites, changing codon identities from Ser to Phe and Arg to Trp, respectively. Mutants *mef26-1* and *mef 26-2* exhibit strong deficits in *COX3* edition but only a 20% decrease in the edition of *NAD4* [172]. Mutant plants in any of these proteins do not exhibit phenotypic defects under normal growth conditions.

The CRM domain is a recently recognized RNA-binding motif of bacterial origin that is present in several group II intron splicing factors. mCSF-1 is a mitochondria-localized CRM family member required for the processing of many mitochondrial introns, and is involved in the biogenesis of respiratory Complexes I and IV in Arabidopsis [88]. Embryo development is arrested during the early globular stage in knock-out *mcsf-1* mutant plants, showing that mCSF-1 is an essential gene. Knock-down *mcsf-1* mutants show germination delay and growth alterations. The respiratory activity is lower in *mcsf-1* plants, and the relative accumulation of native Complexes I and IV is reduced. 

### 5.3. Mutant Plants Indirectly Connected to COX Activity

Mutations in the dual-targeted mitochondrial and plastidial RPOTmp RNA polymerase cause defects in transcript abundance of mitochondrial genes and a dramatic decrease of Complex I and Complex IV levels [91]. *rpoTmp* mutant lines show wrinkly rosette leaves with delayed development from germination onwards [91].

SLO2 (SLOW GROWTH 2) is a PPR protein belonging to the E+ subclass of the PLS subfamily. In *slo2* mutants, seven editing changes producing four amino acid changes in NAD4L (S37L), NAD1 (T1M), NAD7 (L247F) and MTTB (P49S) were detected. These mutants show a marked reduction in Complexes I, III and IV of the mETC. The *slo2* mutants are characterized by retarded leaf emergence, delayed development, late flowering and smaller roots. Sugar, ATP and NAD^+^ contents are lower in *slo2* mutants, and plant phenotype is enhanced in the absence of sucrose, suggesting a defect in energy metabolism [93].

MRPL1 (Mitochondrial Ribosomal Protein L1) is an essential mitochondrial ribosomal protein. Consistent with its role, levels of mitochondrial COX2 are severely reduced in *mrpl1* mutants, whereas no changes are observed in the expression of the nuclear encoded-mitochondrial proteins. *mrpl1-1* and *mrpl1-3* mutants exhibit delayed growth under normal growth conditions, with reduced respiration compared to wild-type plants, whereas the levels of Complex I and Complex IV are strongly reduced [173]. The mito-nuclear protein imbalance induces a proteotoxic stress, thus activating MAPK6 and hormonal (mainly auxin and ethylene) signaling pathways [173].

Mutants in the *Arabidopsis* mitochondrial LON1 protease [95] show a retarded growth phenotype [96]. Mitochondrial proteome characterization of *lon1* mutant plants showed an altered abundance of enzymes of the TCA cycle and OXPHOS [95]. In addition, the *lon1* mutant has lower abundance of Complexes I, IV, and V [95].

Welchen et al. [97] studied the role of CYT*c*, which transfers electrons from Complex III to Complex IV, in plant development and redox homeostasis. In Arabidopsis, CYT*c* is encoded by two genes, designated *CYTC-1* and *CYTC-2*. Knock-out of *CYTC-1* and *CYTC-2* causes embryo developmental arrest indicating that CYT*c* is essential for plant development. Moreover, double T-DNA insertion mutant plants with considerably reduced CYT*c* levels show delayed development, altered expression of stress responsive genes and reduced COX activity [97]. These results suggest that CYT*c* plays an important role in the control of stability of COX in plant mitochondria [97].

## 6. Requirement of COX Activity for Plant Growth and Development

The analysis of mutants suggests that COX activity is important for several aspects of plant development.

### 6.1. Embryogenesis

As we mentioned, COX components are preferentially expressed in plant tissues with high energy demands, like shoot and root meristems, anthers and, significantly, during all stages of embryogenesis (Figure 1). These characteristics suggest that an optimal COX activity may be essential during embryogenesis, in the initial phases of the formation of a new plant. In agreement with this, lack of the COX biogenesis factors AtCOX10 and AtHCC1 originate embryo lethality at various developmental stages, mostly at the heart [18,19] and torpedo [17] stages. There are also several examples of mutants in different COX editing and RNA processing factors, or in proteins connected to Complex IV stability, that also exhibit severe embryo lethality [79,80,87,97,98] (Table 2 for details). 

### 6.2. Germination

Mitochondria are dynamic organelles that constantly change their form and function in response to different external and internal factors, such as organ, tissue, developmental stage, environmental stimuli, and cell energy and metabolic demands, among others ([174,175], and reviewed in [1]). Mitochondria play a crucial role during germination, providing cellular energy via oxidative phosphorylation [175]. Studies performed in maize and rice demonstrated that respiration increases rapidly during the first 24 h in seeds exposed to stratification conditions (dark, 4 °C) [176]. This is due to the execution of a program where the components of the protein import machinery are already present in undifferentiated promitochondrial structures to facilitate a rapid rate of mitochondrial biogenesis after imbibition [177]. In rice, transcripts for two nuclear-encoded COX components (Os*Cox5b-1* and Os*Cox5b-2*) reached maximum levels after 24 h of imbibition, being COX activity detected at 12 h post-imbibition [177]. In Arabidopsis, promitochondria are bioenergetically active immediately upon hydration and respiration increases rapidly within the first hour if evaluated at 21 °C [178]. Law et al. [175] showed the existence of changes in mitochondrial number, size, and morphology after 12 h of imbibition in continuous light and established a triphasic progression model based on the expression profile of genes encoding functional proteins for mitochondrial biogenesis during germination. First, there is an early increase of transcripts associated with nucleic acid metabolism (DNA transcription and RNA editing/processing), followed by transcripts encoding proteins associated with protein synthesis and import and, finally, transcripts encoding proteins of the mitochondrial electron transport chain (mETC) [175,176,177]. Transcripts for components of the mETC reach maximum levels during the transition between promitochondria and mature mitochondria, characterized by a reduced biogenesis and an increase in the bioenergetic and metabolic function of the organelle [175]. This model is in agreement with the data showed in Figure 2H where a clear initial increment in transcripts for RNA processing and cofactor assembly/synthesis proteins can be identified, in comparison with an opposite behavior for the mitochondrial transcripts, when the expression is evaluated in imbibed seeds compared to dry seeds (Figure 2H). Beyond the transcriptional data, there are several examples of Arabidopsis mutants affected in COX composition and activity that exhibit problems and serious delays during the germinative phase [97,88]. In an extreme example, *cod1-1* an *cod1-2* are mutants in an RNA editing factor that lack COX activity and are only able to germinate if embryos from immature seeds are cultivated in a special medium [80] (see below, Table 2)

### 6.3. Vegetative Growth and Development

There are several examples portraying the connection between COX and plant growth and development. Abnormalities or dysfunctions in COX composition and/or activity severely impact on growth and development in Arabidopsis [79,81,82,87,89,98], maize [83] and *Physcomitrella patens* [84,86,90]. Due to lower COX activity, plants exhibit smaller rosettes [73,92,97] with altered rosette leaf shape [91,78], and root growth impairment [69,97]. The *cod1* mutant, with negligible COX activity, exhibits an anarchical slow-growing phenotype leading to a small bush-like structure. Root development is also severely compromised and only sporadically plants are able to produce flowers, with no viable pollen [80] (Table 2).

### 6.4. Senescence

Most mutants in proteins connected with COX activity exhibit delayed growth and development, and this is usually connected to an extended life period [98]. As an opposite example, *Arabidopsis* heterozygous mutants in *AtCOX10*, that possess defective respiration with lower levels of COX activity, exhibit an early onset and an accelerated progression of natural senescence and dark-induced senescence [17]. It was postulated that lower COX activity levels may cause a decrease in energy availability, thus accelerating the use of reserves. Alternatively, dysfunctional COX assembly intermediates may have deleterious effects on the natural progression of the plant developmental program [17]. Further studies, in which COX activity is specifically affected during plant senescence, are required to evaluate the actual requirement of COX for this process.

## 7. Conclusions

COX biogenesis is a modular process controlled by finely regulated steps to assemble a functional respiratory complex for the successful operation of the mETC. Expression of proteins encoded in two genomes must be coordinated, including those responsible for COX catalytic activity, for the synthesis and delivery of redox-active metal centers and prosthetic groups, for the assembly of subunits, and for the multiple RNA processing events. COX has several plant-specific subunits and, while mitochondria-encoded, catalytic-core subunits are extremely conserved, homologs of several yeast and human nuclear-encoded subunits are missing in plants. In a similar way, COX biogenesis proteins related to the assembly of the redox-active centers are conserved in plants, but other COX assembly factors are not. Plant COX assembly factors also seem to have acquired novel functions, not directly related to COX assembly. The presence of alternative isoforms of certain subunits suggests that COX composition is finely regulated according to cell- and tissue-type, and plant developmental stage. This may establish COX complexes with different properties, able to adjust COX function to energy demands. In agreement with the connections between mitochondrial respiratory activity and energy demands for growth, expression of COX components is regulated at the transcriptional level by nutrients and the diurnal cycle. In addition, several energy-demanding processes, such as embryogenesis and germination, also generate an increase in the expression of the majority of COX-related genes. COX activity is also under hormonal control and there are several pieces of evidence for possible regulation at the transcriptional and post-translational levels. A decrease in COX activity produces strong alterations of plant growth and development, often leading to embryonic lethality. This can be thought of as a double-check control mechanism, where hormones regulate COX biogenesis and growth, and COX is directly connected to plant growth and development through its activity. Finally, several COX assembly factors were recognized as stress-related proteins, acting in response to abiotic and biotic stress in plants. As we previously speculated, COX assembly factors or structural proteins may accumulate under stress to avoid or diminish ROS generation due to dysfunctional COX and/or to actively replace damaged COX subunits. Much less is known about COX biogenesis in plants than in yeast and mammals. Further work is needed to clarify the role and significance of plant-specific subunits, to identify putative plant-specific COX assembly factors, to unequivocally establish the interactions between the different proteins involved in COX biogenesis, and to elucidate if the stress-related functions of several accessory proteins are related to COX activity or more widely connected to retrograde signaling and other mitochondrial regulatory pathways. 

## Figures and Tables

**Table 1:** COX subunits and assembly factors identified in yeast, humans and Arabidopsis.

Yeast (*S. cerevisiae*)	Humans		*Arabidopsis*	
		Role described in yeast and/or mammals		AGI
Mitochondrial catalytic-core subunits				
Cox1	COX1	Cytochrome *c* oxidase subunit 1	COX1	ATMG01360
Cox2	COX2	Cytochrome *c* oxidase subunit 2	COX2	ATMG00160
Cox3	COX3	Cytochrome *c* oxidase subunit 3	COX3	ATMG00730
				AT2G07687
Structural nuclear subunits				
Cox4	COX5b		COX5b-1	AT3G15640
			COX5b-2	AT1G80230
			COX5b-3	AT1G52710
Cox5a	COX4-1, COX4-2 ^a^		-	-
Cox5b ^b^	-		-	-
Cox6	COX5A		-	-
Cox7	COX7A1, COX7A2 ^a^	Required for COX assembly and function	-	-
Cox8	COX7C		-	-
-	COX8-1, COX8-2, COX8-3 ^a^		-	-
Cox9	COX6C, COX7B, COX8A, COX8B		-	-
COX12/COXVIb	COX6B1, COX6B2 ^a^	Not essential for COX assembly or function	COX6b-1	AT1G22450
			COX6b-2	AT5G57815
			COX6b-3	AT4G28060
			COX6b-4	AT1G32710
Cox13/CoxVIa	COX6A1 ^a^ COX6A2 ^a^		COX6a	AT4G37830
-	-		COX5c-1	AT2G47380
-	-		COX5c-2	AT3G62400
-	-		COX5c-3	AT5G61310
-	-		COX-X1-1	AT5G27760
-	-		COX-X2-1	AT4G00860
-	-		COX-X2-2	AT1G01170
-	-		COX-X3	AT1G72020
-	-		COX-X4	AT4G21105
-	-		COX-X5	AT3G43410
-	-		COX-X6	AT2G16460
COX Assembly Factors				
		Role described in yeast and/or mammals		AGI
Membrane insertion and processing of catalytic-core subunits				
Oxa1	OXA1L	Mitochondrial insertase, mediates insertion of COX subunits into the IMM	OXA1 ^d^	AT5G62050
			OXA1L ^d^	AT2G46470
Cox20	COX20	COX2 chaperone for copper metalation	-	-
Cox18	COX18	Translocation and export of the COX2 C-terminal tail into the IMS	-	-
Mss2	-	Peripherally bound IMM protein of the mitochondrial matrix; involved in membrane insertion of COX2 C-terminus ^b^	-	-
Pnt1	-	Export of the COX2 C-terminal tail ^b^	-	-
Imp1	IMMP1	Catalytic subunit of the IMM peptidase complex; required for maturation of mitochondrial proteins of the IMS	Peptidase S24/S26A/S26B/S26C	AT1G53530
				AT1G29960
				AT1G23465
Imp2	IMMP2	Required for the stability and activity of Imp1	MYB3R-3	AT3G08980
-	NDUFA4 NDUFA4L2	Assembly factor for COX or supercomplexes in mitochondria of growing cells and cancer tissues ^c^	-	-
Heme A Biosynthesis and Insertion				
Cox10	COX10	Farnesylation of heme B	COX10	AT2G44520
Cox15	COX15	Heme A synthase required for the hydroxylation of heme O to form heme A	COX15	AT5G56090
Yah1	FDX2	Collaborates with COX15 in heme O oxidation. Essential for heme A and Fe/S protein biosynthesis	MFDX1	AT4G05450
			MFDX2	AT4G21090
Ahr	ADR	Collaborates with COX15 in heme O oxidation Pyridine nucleotide-disulphide oxidoreductase family protein	MFDR	AT4G32360
Shy1	SURF1	Required for efficient COX assembly in the IMM. Involved in a step of COX1 translation and assembly; proposed to participate in heme A delivery	SURF1-1	AT3G17910
			SURF1-2	AT1G48510
Copper Trafficking and Insertion				
Sco1	SCO1	Copper chaperone, transporting copper to the CuA site on COX2	HCC1	AT3G08950
Sco2	SCO2		HCC2	AT4G39740
Coa6	COA6	Cooperates with SCO2 in the metalation of CuA	COA6-L	AT5G58005
Cox11	COX11	Assembly of CuB in COX1	COX11	AT1G02410
Cox17	COX17	Copper metallochaperone that transfers copper to SCO1 and COX11	COX17-1	AT3G15352
			COX17-2	AT1G53030
Cox19	COX19	Interacts with COX11 as a reductant, critical for COX11 activity	COX19-1	AT1G66590
			COX19-2	AT1G69750
Cox23	COX23	COX assembly factor, unknown function	COX23	AT1G02160
				AT5G09570
Pet191	PET191	Protein required for COX assembly; contains a twin CX9C motif; imported into the IMS via the MIA import machinery	PET191	AT1G10865
Cmc1	CMC1	Stabilizes the COX1-COX14-COA3 complex prior to the incorporation of subunits COX4 and COX5a. Maintains COX1 in a maturation-competent state before insertion of its prosthetic groups	CMC1	AT5G16060
Cmc2	CMC2	COX biogenesis protein	CMC2	AT4G21192
Mir1 Pic2	SLC25A3	Mitochondrial copper and phosphate carrier; imports copper and inorganic phosphate into mitochondria.	PHT3-1 PHT3-2 PHT3-3	AT5G14040 AT3G48850 AT2G17270
Mrs3	MITOFERRIN-1 MITOFERRIN-2	Iron transporter; mediates Fe^2+^ and copper transport across the IMM; mitochondrial carrier family member	Mitochondrial substrate carrier protein	AT1G07030 AT2G30160 AT5G42130
Cox Assembly (other)				
Cox14	COX14 (C12orf62)	Involved in translational regulation of COX1, avoiding COX1 aggregation before assembly	-	-
Cox16	COX16	Mitochondrial IMM protein; required for COX assembly	COX assembly protein	AT4G14145
Cox24	-	Mitochondrial IMM protein; required for accumulation of spliced COX1 mRNA ^b^	-	-
Cox26	-	Stabilizes the formation of Complex III-IV supercomplexes ^b^	-	-
Coi1	-	Interacts with subunits of Complexes III and IV. Essential for supercomplex formation ^b^	-	-
Rcf1	HIGD1A	Stabilizes the COX4-COX5A module and promotes its assembly with COX1 ^c^	ATL48	AT3G48030
	HIGD2A	Supports the formation of a class of COX-containing supercomplexes ^c^		
Rcf2	-	Required for late-stage assembly of the COX12 and COX13 subunits and for COX activity ^b^	ATHIGD3	AT3G05550
Coa1	COA1/MITRAC15	Required for assembly of Complex I and Complex IV in mammals. Interacts with Shy1 during the early stages of assembly in yeast	-	-
Coa2	-	Acts downstream of assembly factors Mss51 and Coa1 and interacts with assembly factor Shy1 ^b^	-	-
-	COA3/MITRAC12	Required for efficient translation of COX1 ^c^	-	-
Coa3/Cox25	-	Required for COX assembly; involved in translational regulation of COX1 and prevention of COX1 aggregation before assembly ^b^	-	-
Coa4	COA4	Twin CX9C protein involved in COX assembly and/or stability		
-	MITRAC7	Chaperone-like assembly factor required to stabilize newly synthesized COX1 and to prevent its premature turnover ^c^	-	-
-	MR-1S	Short isoform of the myofibrillogenesis regulator 1 (MR-1S). Interacts PET100 and PET117 chaperones	-	-
	TMEM177	TMEM177 associates with newly synthesized COX2 and SCO2 in a COX20-dependent manner		
Mss51	-	*COX1* mRNA-specific translation activator. Influences COX1 assembly into COX ^b^	-	-
Mss18	-	Required for efficient splicing of mitochondrial *COX1* ^b^	-	-
Mba1	-	Membrane-associated mitochondrial ribosome receptor; possible role in protein export from the matrix to the IMM ^b^	-	-
Mne1	-	Involved in *COX1* mRNA intron splicing ^b^	-	-
Mrs1	-	Splicing protein; required for splicing of two mitochondrial group I introns ^b^	-	-
Mrp1	-	Mitochondrial ribosomal proteins specific for *COX2* and *COX3* mRNA ^b,e^	-	-
Mrp17	-		-	-
Mrp21	-		-	-
Mrp51	-		-	-
Mrpl36	-		-	-
Pet54	-	Protein required to activate translation of the *COX3* mRNA, to process the aI5β intron on the *COX1* transcript, and required for Cox1 synthesis ^b^	-	-
Pet100	PET100	Chaperone that facilitates COX assembly	-	-
Pet111	-	Mitochondrial translational activator specific for the *COX2* mRNA ^b^	-	-
Pet117	PET117	Assembly factor that couples heme A synthesis to Complex IV assembly	-	-
Pet122	-	Mitochondrial translational activator specific for the *COX3* mRNA ^b^	-	-
Pet123	-	Mitochondrial ribosomal protein of the small subunit ^b^	-	-
Pet494	-	Mitochondrial translational activator specific for the *COX3* mRNA ^b^	-	-
Pet309	LRPPRC	Specific translational activator for the COX1 mRNA	PPR superfamily protein ^d^	AT2G02150
				AT1G52640
				AT5G16640
Dcp29	TACO1	Translational activator of mitochondria-encoded COX1	-	-
Oma1	OMA1	Metalloendopeptidase that is part of the quality control system in the IMM; important for respiratory supercomplex stability	MIO24.13	AT5G51740
Mam33	C1QBP	Specific translational activator for the mitochondrial *COX1* mRNA	MAM33-L Mitochondrial glycoprotein family	AT5G02050
				AT1G80720
				AT1G15870
				AT3G55605
				AT5G05990
				AT2G39795
				AT4G31930
				AT2G41600
Ssc1/HSP70	HSPA9	Facilitates translational regulation of COX biogenesis	mtHSC70-1	AT4G37910
			mtHSC70-2	AT5G09590
AI1/Q0050	-	Intron maturase; type II family protein	MATR	ATMG00520
AI2/Q0055	-			

^a^ Mammalian tissue specific variants (COX4-1, COX6A1, COX6B1, COX7A2, COX8-2: Liver/Ubiquitous; COX4-2: Lung; COX6A2, COX7A1, COX8-1: Heart; COX6B2, COX8-3: Testis). ^b^ Function described only in yeast. ^c^ Function described only in mammals. ^d^ Only proteins with the highest sequence identity were included (*p*-value < 10^−8^ for PPR family proteins and *p*-value < 10^−20^ for OXA-related proteins). ^e^ Information from the yeast genome database. AGI: *Arabidopsis* genome initiative.

**Table 2:** Mutants that exhibit COX altered composition and/or activity.

COX Assembly Factors						
Affected Process	Name/AGI	Mutant Name/Code	Description	Phenotype	Complex IV Accumulation/Respiration	Ref.
Copper delivery and insertion	HCC1 AT3G08950	*hcc1-1 SALK_057821* *hcc1-2* *GABI-Kat923A11*	Homolog of the yeast Copper Chaperone Sco1	Embryos are arrested at various developmental stages. Altered response of root elongation to copper	Very low levels of COX activity in embryos and rosette leaves	[18,19]
	HCC2 AT4G39740	*hcc2-1* *GABI-Kat843H01 hcc2-2* *GABI-at640A10*	Homolog of the yeast Copper Chaperone Sco1	Knockout lines exhibit only mild growth suppression. More sensitive to UV-B treatment	Normal COX activity	[18,20]
	COX17-1 AT3G15352 COX17-2 AT1G53030	*cox17-2* *SALK_062021C* + *amiR-COX17-1*	Cytochrome *c* oxidase 17, a soluble protein from the IMS that participates in the transfer of copper to COX	Smaller rosettes and roots. Severity of the phenotype is related with the decrease in the level of COX17	Slight decrease in COX activity in *cox17-2* mutants	[73]
	COX11 AT1G02410	*KD1* *SALK_105793* *KD2* *SALK_003445C*	Cytochrome *c* oxidase 11, involved in copper delivery to COX	Defects in pollen germination, root growth inhibition, smaller rosettes and leaf curling	Reduced COX activity	[69]
Heme A synthesis	COX10 AT2G44520	*cox10* *SAIL_1283_D03.V1*	Homolog to the yeast farnesyltransferase Cox10 that catalyzes the conversion of heme B to heme O in the heme A biosynthesis pathway	Homozygous mutants are embryo-lethal. Heterozygous mutant plants show early onset and progression of natural and dark-induced senescence	Reduced COX activity. Normal levels of total respiration; lower levels of cyanide-sensitive respiration, increased AOX respiration.	[17]
RNA Processing Factors ^a^						
Affected Process	Name/AGI	Mutant Name and Code	Description	Phenotype	Complex IV Accumulation/Respiration	Ref.
Editing	RIP1/MORF8 AT3G15000	*rip1/morf8* *FLAG_150D11*	Lacks the PRR motif. Interacts with the chloroplast-PPR protein RARE1	Dwarf phenotype	NA	[78]
	MORF1 AT4G20020	*morf1* WiscDsLox419C10	Required for RNA editing in plant mitochondria	Abortion of homozygous mutant seeds	NA	[79]
	COD1 AT2G35030	*cod1-1 (SALK_000882)* *cod1-2 (SALK_615308)*	Mitochondria-localized PLS-subfamily PPR protein	Germination deficiency; shoot and root growth retardation. No viable pollen	Absence of COX activity	[80]
	MEF13 AT3G02330	*mef13-1* *EMS mutant* *mef13-2/*SALK_097270C	E-type PPR protein required for editing at 8 sites in Arabidopsis. Dual targeted protein	Growth retardation	NA	[81]
	ORRM4 AT1G74230	*orrm4* *SALK_023061C*	Organelle RNA Recognition Motif-containing protein	Vegetative growth and flowering retardation	NA	[82]
	DEK10 *Zea mays*	*dek10/dek10-N1176A*	Defective kernel 10; encodes an E-subgroup PPR protein in maize	Small kernels and vegetative growth delay	Reduced Complex IV activity and COX2 accumulation	[83]
	ppPPR_78/*Physcomitrella patens*	*ΔppPPR_78*	PPR protein family member	Slight growth retardation	NA	[84,85]
	ppPPR_77/*Physcomitrella patens*	*ΔppPPR_77*	PPR protein family member	Severe growth retardation	NA	[86]
Splicing	WTF9 AT2G39120	*wtf9-1* *SALK_022250* *wtf9-2* *SALK_143433*	Protein involved in the splicing of group II introns in mitochondria	Severely stunted shoots and roots. Small flowers, small anthers and little pollen. Aborted seeds	Reduced COX2 accumulation	[87]
	mCSF-1 AT4G31010	*mcsf-1* SALK-086774	Mitochondria-localized CRM family member required for the processing of many mitochondrial introns	Germination delayed. Altered growth pattern and delayed development	Reduced Complex I and Complex IV activity. Reduced levels of COX2 and decrease of fully-assembled COX. Reduced respiration	[88]
	mMAT2 AT5G46920 PMH2 AT3G22330	*Double mutant nmat2 x pmh2* *SALK-064659 x SAIL-628C06*	Members of the maturase and RNA helicase families; function in the splicing of many introns in Arabidopsis mitochondria	Growth delay and alterations in vegetative and reproductive development	Less sensitive to KCN (COX inhibitor)	[89]
	ppPPR_43/*Physcomitrella patens*	*ΔppPPR_43*	Involved in the splicing of *COX1*	Severe growth retardation	NA	[90]
Other Mutants Affected in Cox Assembly/Activity						
Affected Process	Name/AGI	Mutant Name and Code	Description	Phenotype	Complex IV Accumulation/Respiration	Ref.
Mitochondrial transcription	RPOTmp AT5G15700	*rpoTmp-1 GABI_286E07* *rpoTmp-2* *SALK_132842*	T3/T7 phage-type dual-targeted RNA polymerase	Delayed plant development, wrinkly rosette leaves	Reduced abundance of the respiratory Complexes I and IV	[91,92]
RNA processing	SLO2 AT2G13600	*slo2-2* *SALK_021900* *slo2-3* *Tilling line T94087*	PPR protein belonging to the E+ subclass of the PLS subfamily	Retarded leaf emergence, delayed development, late flowering and smaller roots	Marked reduction in Complexes I, III, and IV	[93]
Mitochondrial translation	MRPL1 AT2G42710	*mrpl1-1 SALK_206492C* *mrpl1-3 SALK_014201*	Mitochondrial Ribosomal Protein L1.	Delayed plant growth	Reduced levels of Complexes I and IV. COX2 protein is severely reduced. Reduced respiration	[94]
Protein processing	LON1 AT5G26860	*lon1-1* *EMS-line* *lon1-2* *SALK_012797*	ATP-dependent protease and chaperone	Retarded growth of both shoots and roots	Lower abundance of Complexes I, IV, and V	[95,96]
Complex IV abundance and stability	CYTC-1 AT1G22840 CYTC-2 AT4G10040	*cytc-1a GK586B10* *cytc-1b* *SALK_143142* *cytc-2a SALK_037790* *cytc-2b* *SALK_029663*	Cytochrome *c* (CYT*c*) is an IMS electron carrier that transfers electrons between Complexes III and IV	Knock-out of both genes produces embryo-lethality. Plants exhibit smaller rosettes with a pronounced decrease in parenchymatic cell size and a delay in plant development	Decreased levels of Complex IV. Normal levels of total respiration; lower levels of cyanide-sensitive respiration and increased AOX-respiration	[97]

^a^ Part of the information contained in this table was extracted from Colas des Francs-Small C. and Small I. [98]. NA: not analyzed.

## Supplementary Materials

Supplementary materials can be found at http://www.mdpi.com/1422-0067/19/3/662/s1.

## Abbreviations

COX	Cytochrome *c* oxidase
IMM	Inner mitochondrial membrane
IMS	Intermembrane space
mETC	Mitochondrial electron transport chain
MS	Mass spectrometry
OXPHOS	Oxidative phosphorylation
PTMS	Posttranslational modifications
