# Supplementary material for: The Complexity of Mitochondrial Complex IV: An Update of Cytochrome c Oxidase Biogenesis in Plants
Source: Int J Mol Sci. 2018 Feb 27;19(3):662. doi: 10.3390/ijms19030662 (PMC5877523; doi:10.3390/ijms19030662)
Supplement: Supplementary file 1 [file ijms-19-00662-s001.zip › ijms-265590-supp-final.docx]

**Supplementary Materials: The Complexity of Mitochondrial Complex IV: An Update of Cytochrome *c* Oxidase Biogenesis in Plants**

**Natanael Mansilla, Sofia Racca, Diana E. Gras, Daniel H. Gonzalez and Elina Welchen**

**Table S1.** COX subunits and assembly factors in *Arabidopsis thaliana*.

| **Mitochondria-Encoded Catalytic-Core Subunits** | | | | | | | | |
| --- | --- | --- | --- | --- | --- | --- | --- | --- |
|  | **AGI** | **Prot ID** | **Protein Name** | **Description** | **Experimental Evidence ^a^** | **PTMS ^b^** | **PPI ^c^** | **Ref. ^d^** |
| COX1 | ATMG01360 | P60620 | Cytochrome *c* oxidase subunit 1. | COX is the component of the respiratory chain that catalyzes the reduction of oxygen to water. Subunits 1–3 form the functional core of the enzyme complex. Electrons originating in CYTc are transferred via the Cu_A_ center of COX2 and heme *a* of subunit 1 to the bimetallic center formed by heme *a*_3_ and Cu_B_. | MS [1] | NR | NR | [2] |
| COX2 | ATMG00160 | P93285 | Cytochrome *c* oxidase subunit 2. |  | MS [3–9] | NR | NR | [2,10] |
| COX3 | ATMG00730 | P92514 | Cytochrome *c* oxidase subunit 3. |  | MS [1] | NR | NR | [2] |
| **Nuclear-Encoded Structural Subunits** | | | | | | | | |
|  | **AGI** | **Prot ID** | **Protein Name** | **Description** | **Experimental Evidence ^a^** | **PTMS ^b^** | **PPI ^c^** | **Ref. ^d^** |
| COX5b-1 | AT3G15640 | Q9LW15 | Cytochrome *c* oxidase subunit 5b-1. | Nuclear-coded COX subunit. | MS [1,3–9]  TP: 1-55 [11] | P-3 [12,13] | AT3G02150 [14] | [15–18] |
| COX5b-2 | AT1G80230 | Q9SSB8 | Cytochrome *c* oxidase subunit 5b-2. | Nuclear-coded COX subunit. | MS [1,3–9]  TP: 1-54 [11] | Ubi-78 [19] | NR | [15,16,20] |
| COX5b-3 | AT1G52710 | Q9SSS5 | Putative cytochrome *c* oxidase subunit 5b-3. | Rubredoxin-like superfamily protein; nuclear-coded COX subunit. | Mitochondrion: 1.0 [21] | NR | NR | NR |
| COX5c-1 | AT2G47380 | O22912 | Cytochrome *c* oxidase subunit 5c-1. | Nuclear-coded COX subunit. | MS [1,4,6,9,10] | NR | NR | [10,22–24] |
| COX5c-2 | AT3G62400 | Q9LZQ0 | Cytochrome *c* oxidase subunit 5c-2. | Nuclear-coded COX subunit. | MS [9] | NR | NR | [22–24] |
| COX5c-3 | AT5G61310 | Q9FLK2 | Probable cytochrome *c* oxidase subunit 5c-3. | Nuclear-coded COX subunit. | MS [9] | NR | NR | [22–24] |
| COX6a | AT4G37830 | Q9T070 | Cytochrome *c* oxidase subunit 6a. | Nuclear-coded COX subunit. | FP [25]  MS [1,4,9,10]  TP: 1-36 [26] | P-1 [12] | NR | [27] |
| COX6b-1 | AT1G22450 | Q9S7L9 | Cytochrome *c* oxidase subunit 6b-1. | Nuclear-coded COX subunit. | MS [4,5,7–9,28] | N-Ace: 23, 160 [28,29]  DIS 137-169, 147-158 [26] | AT5G35750 [30] | [10,27,31–33] |
| COX6b-2 | AT5G57815 | Q94SL0 | Cytochrome *c* oxidase subunit 6b-2. | Nuclear-coded COX subunit. | Mitochondrion: 1.0 [21] | NR  DIS 25-57, 35-46 [26] | NR | [31,33] |
| COX6b-3 | AT4G28060 | Q9SUD3 | Cytochrome *c* oxidase subunit 6b-3 | Nuclear-coded COX subunit. | MS [8–10] | NR | NR | [31,33] |
| COX6b-4 | AT1G32710 | Q9LPJ2 | Putative cytochrome *c* oxidase subunit 6b-4. | COX subunit VIb family protein. | Mitochondrion: 1.0 [21] | DIS 74-106, 84-95 [26] | NR | NR |
| COX-X1 | AT5G27760 | Q8LG60 | Hypoxia-responsive family protein (ATHIG2) | Integral component of the IMM, as a part of Complex IV. | MS [1,6,9] | NR | AT1G35670 [34] | [35] |
| COX-X2-1 | AT4G00860 | Q38842 | ATOZI1 | Putative pathogenesis-related protein induced in response to ozone, *Pseudomonas* strains and ROS. | MS [1,4–9] | UBI-76 [19] | NR | NR |
| COX-X2-2 | AT1G01170 | Q2HIQ2 | Ozone-responsive stress-like protein (DUF1138) | Integral membrane component, involved in stress response. | MS [8–10] | NR | NR | NR |
| COX-X3 | AT1G72020 | Q9C7G5 | TonB-dependent heme receptor A | Integral membrane component. | MS [1] | NR | NR | NR |
| COX-X4 | AT4G21105 | Q944S8 | Cytochrome c oxidase/electron carrier | Integral membrane component. | MS [1,8–10] | UBI-2,16 [19] | AT3G57040 [36] | NR |
| COX-X5 | AT3G43410 | Q9M178 | F-box/LRR protein. | Protein of unknown function. | MS [1,4] | NR | NR | NR |
| COX-X6 | AT2G16460 | Q9SIV6 | Coiled-coil 90B-like protein (DUF1640) | Protein of unknown function (DUF1640) | MS [1,4,6,7–9,10,37]  TP: 1-43 [26] | NR | NR | NR |
| **COX Assembly Factors** | | | | | | | | |
| ***Membrane Insertion and Processing of Catalytic Core Subunits*** | | | | | | | | |
|  | **AGI** | **Prot ID** | **Protein Name** | **Description** | **Experimental Evidence ^a^** | **PTMS ^b^** | **PPI ^c^** | **Ref. ^d^** |
| OXA1 | AT5G62050 | Q42191 | IMM protein OXA1 (Oxidase assembly 1 protein). | Membrane insertion of COX subunits; homologue of yeast oxidase assembly 1 (Oxa1), essential factor for protein sorting and assembly into membranes, essential for COX assembly and activity. | FP [38]  MS [6,9] | NR | NR | [39] |
| OXA1L | AT2G46470 | Q9SKD3 | IMM protein OXA1-like. | Probably required for the insertion of integral membrane proteins into the IMM. | MS [6,9]  TP: 1-22 [26] | NR | NR | NR |
| Peptidase S24/S26A/S26B/S26C family protein | AT1G23465 | Q5BIV4 | Peptidase S24/S26A/S26B/S26C family protein. | IMM peptidase complex; serine-type peptidase activity. | Mitochondrion: 1.0 [21] | NR | NR | NR |
|  | AT1G53530 | Q6NLT8 |  |  | MS [9,37] | NR | NR | NR |
|  | AT1G29960 | Q67XF2 |  |  | MS [9] | NR | NR | NR |
| MYB3R-3 | AT3G08980 | Q9S724 |  |  | Mitochondrion: 1.00 [21] | NR | NR | [26] |
| ***Heme A Biosynthesis and Insertion*** | | | | | | | | |
|  | AGI | Prot ID | Protein Name | Description | Experimental evidence ^a^ | PTMS ^b^ | PPI ^c^ | Ref. ^d^ |
| COX10 | AT2G44520 | O64886 | Protoheme IX farnesyltransferase, mitochondrial. | Heme O synthase. Converts protoheme IX and farnesyl diphosphate to heme O. Essential protein for COX assembly and activity. | FP [40]  MS [37]  TP: 1-33 [26] | NR | NR | [40] |
| COX15 | AT5G56090 | Q9FKT8 | Cytochrome *c* oxidase 15. | Involved in the biosynthesis of heme A, oxidation of heme O methyl to formyl; transcriptionally increased after treatment with rotenone. | MS [7,9] | NR | NR | NR |
| MFDX1 | AT4G05450 | Q9M0V0 | Adrenodoxin-like protein 1. | Collaborates with COX15 in heme O oxidation. Associates in vitro with the adrenodoxin reductase MFDR to form an efficient low potential electron transfer chain that is able to reduce CYTc. | FP [41]  MS [8]  TP: 1-35 [26] | NR | NR | [41,42] |
| MFDX2 | AT4G21090 | Q8S904 | Adrenodoxin-like protein 2. |  | FP [41]  TP: 1-74 [26] | UBI-217 [19] | NR | NR |
| MDFR | AT4G32360 | Q8W3L1 | Mitochondrial ferredoxin reductase. | Associates *in vitro* with the adrenodoxin-like protein MFDX1 to form an efficient low potential electron transfer chain that is able to reduce CYTc. | FP [41]  MS [6,9]  TP: 1-14 [26] | NR | NR | [41,42] |
| SURF1-1 | AT3G17910 | Q9SE51 | SURFEIT 1 | Similar to human SURF1 which is known to be involved in COX assembly. Catalyzes an assembly step involving COX1; proposed to participate in heme A delivery. | MS [4,6,7,9,10] | NR | NR | NR |
| SURF1-2 | AT1G48510 | Q9LP74 | Surfeit locus 1 cytochrome c oxidase biogenesis protein. | Probably involved in the biogenesis of the COX complex. | Mitochondria: 1.0 [21] | NR | AT1G76310  AT3G10525 [43] | NR |
| ***Coper Trafficking and Insertion*** | | | | | | | | |
|  | **AGI** | **Prot ID** | **Protein Name** | **Description** | **Experimental Evidence ^a^** | **PTMS ^b^** | **PPI ^c^** | **Ref. ^d^** |
| HCC1 | AT3G08950 | Q8VYP0 | Electron transport SCO1. | Homologue of the copper chaperone Sco1 from the yeast *S. cerevisiae*. SCO1 encodes a mitochondrial protein that is essential for COX assembly. Thought to play a role in cellular copper homeostasis, mitochondrial redox signaling or insertion of copper into the active site of COX. Plays an essential role in embryo development. | FP [44]  MS [6,9,37]  TP: 1-13 [26] | N-Ace: 314  P-3 | AT1G01910 [14] | [44,45] |
| HCC2 | AT4G39740 | Q8LAL0 | Thioredoxin superfamily protein. | Encodes HCC2, resulting from a gene duplication during plant evolution. With sequence homology to Sco1 proteins, involved in copper insertion during COX assembly in other organisms. HCC2, which lacks the cysteines and histidine putatively involved in copper binding, functions in copper sensing and redox homeostasis. | FP [44]  MS [9]  TP: 1-14 [26] | NR | NR | [44–46] |
| COA6-L | AT5G58005 | Q8RXH9 |  | COX subunit VIb family protein. | MS [9] | UBI-21 [19] | NR | NR |
| COX11 | AT1G02410 | Q8GWR0 | Cytochrome *c* oxidase assembly protein CtaG/Cox11 family. | Involved in the insertion of Cu_B_ into subunit COX1. | FP [47]  MS [37]  TP: 1-80 [26] | NR | AT4G09570 [34] | [47] |
| COX17-1 | AT3G15352 | Q9LJQ9 | Cytochrome *c* oxidase 17. | Encodes a protein similar to yeast Cox17, a copper-binding protein that mediates the delivery of copper for COX assembly. Functionally complements the yeast *cox17* null mutant. | FP [48]  MS [28] | Ace-28 [28]  DIS 37-66, 47-56 [26]  P-1 [49] | NR | [48,50–52] |
| COX17-2 | AT1G53030 | Q94FT1 | Cytochrome *c* oxidase 17. | Encodes a protein similar to yeast Cox17, a copper-binding protein that mediates the delivery of copper for COX assembly. Functionally complements the yeast *cox17* null mutant. | MS [9] | DIS 35-64, 45-54 [26] | NR | [48,50–52] |
| COX19-1 | AT1G66590 | F4IEX1 | Cytochrome *c* oxidase 19-1. | Cytochrome *c* oxidase 19-1. Functionally complements the yeast *cox19* null mutant. | Mitochondrion: 1.0 [21] | NR | NR | [53] |
| COX19-2 | AT1G69750 | Q9C9L6 | Cytochrome *c* oxidase 19-2. | Cytochrome *c* oxidase 19-2. Functionally complements the yeast *cox19* null mutant. | MS [9] | NR | NR | [53] |
| COX23 | AT1G02160 | Q8VZ44 | Cox19 family protein (CHCH motif). | Cox19 family protein (CHCH motif) | MS [9] | UBI-4 [19] | NR | NR |
| COX23L | AT5G09570 | Q9LXC2 | Cox19-like CHCH family protein. |  |  | NR | NR | NR |
| PET191 | AT1G10865 | Q8GWM1 | Cytochrome *c* oxidase assembly factor. |  | Mitochondrion [26] | UBI-55 [19] | NR | NR |
| CMC1 | AT5G16060 | Q9LFR9 | Cytochrome *c* oxidase assembly factor |  | MS [9] | Ace-23 [29] | AT1G22920 [14] | NR |
| CMC2 | AT4G21192 | Q0WSU0 | Cytochrome *c* oxidase biogenesis protein; Cmc1-like protein |  | Mitochondrion [26] | UBI-4,64 [19] | NR | NR |
| PHT3-1 | AT5G14040 | Q9FMU6 | Mitochondrial phosphate carrier protein. | Transport of phosphate from the cytosol to the mitochondrial matrix. Mediates salt stress tolerance through an ATP-dependent pathway and via modulation of gibberellin metabolism. | MS [4–10] | NR | AT5G43980 [54]  AT3G51030 [55] | [10,39,56–58] |
| PHT3-2 | AT3G48850 | Q9M2Z8 |  |  | FP [59]  MS [4,6,9,28,37] | NR | NR | [39,57,58] |
| PHT3-3 | AT2G17270 | Q7DNC3 |  |  | Mitochondrion: 0.91 [21] | NR | NR | NR |
| Mrs3-like | AT1G07030 | Q8L6Z2 | Mitochondrial substrate carrier family protein. | Integral component of the IMM; iron ion transmembrane transporter activity; iron ion homeostasis; mitochondrial iron ion transport. | MS [9,37] | N-Ace [29] | NR | NR |
|  | AT2G30160 | O64731 |  |  | MS [9] | NR | NR | NR |
|  | AT5G42130 | Q9FHX2 | MITOFERRIN-LIKE 1 |  | MS [37] | NR | NR | [60] |
| ***Cox Assembly (Other)*** | | | | | | | | |
|  | **AGI** | **Swiss-Prot ID** | **Protein Name** | **Description** | **Experimental Evidence ^a^** | **PTMS ^b^** | **PPI ^c^** | **Ref. ^d^** |
| COX assembly protein | AT4G14145 | A0JPW4 | Cytochrome c oxidase assembly protein. | Cytochrome c oxidase assembly protein. | MS [61] | NR | NR | NR |
| ATL48 | AT3G48030 | Q7X843 | Hypoxia-responsive family protein. | Protein modification; protein ubiquitination. | MS [9] | P-1 [49]  Ace [29] | NR | [62] |
| ATHIGD3 | AT3g05550 | Q9M9W0 | Hypoxia-responsive family protein. |  | MS [35] | NR | NR | [35] |
| EMB2794 | AT2G02150 | P0C894 | Putative pentatricopeptide repeat-containing protein | Tetratricopeptide repeat (TPR)-like superfamily protein. | Mitochondrion: 0.91 [21] | NR | NR | [63] |
| PPR superfamily protein | AT1G52640 | Q9SSR6 | Pentatricopeptide repeat (PPR) superfamily protein. |  | Mitochondrion: 0.91 [21,26] | P-1 [12] | NR | [63] |
|  | AT5G16640 | Q9FMD3 | Pentatricopeptide repeat (PPR) superfamily protein |  | Mitochondrion: 0.91 [21,26] | NR | NR | NR |
| RFP2 | AT1G62670 | Q9SXD1 | 5′-mRNA PPR processing protein | Involved in the formation of COX3 mRNA 5’ ends. | Mitochondrion: 1.0 [21] | NR | AT4G26630 [64] | [63,64] |
| WTF9 | AT2G39120 | Q9ZUZ6 | Ubiquitin carboxyl-terminal hydrolase family protein | Involved in the splicing of group II introns in mitochondria. | FP [65] | NR | NR | [66] |
| RIP1/MORF8 | AT3G15000 | Q9LKA5 | Multiple organellar RNA editing factor. | Involved in organellar RNA editing. Required for the processing of numerous RNA editing sites in mitochondria and plastids. | FP [67,68]  MS [6–10]  Dual targeting to mitochondria and chloroplasts [67,68] | NR | AT3G27960 [14] AT5G10270 AT5G64960 [69] MEF10 [71], PPOX1 [72], MORF1/RIP8; MORF2/RIP2; MORF3/RIP3; MORF4/RIP4; MORF5/RIP5; MORF6/RIP6 MORF7/RIP7, PMD1 [73], RBG3/ORRM3RBG5/ORRM4 [74]ORRM1, VAT3/OZ1 [75] MEF35 [76], ORRM6 [77] | [67,68,70] |
| MORF1 | AT4G20020 | O49429 | Multiple organellar RNA editing factors. | Required for RNA editing in plant mitochondria and provides additional components of the RNA editing machinery in plant organelles. | FP [68]  MS [7,9] | NR | MEF35 [76] MEF13 [78] | [79] |
| MEF21 | AT2G20540 | Q9SIL5 | Pentatricopeptide repeat-containing protein. | Required for RNA editing in plant mitochondria. | Mitochondrion: 1.0 [21] | NR | AT4G20020  AT2G33430  AT1G11430 [78] | [63,80] |
| MEF26 | AT3G03580 | Q9SS60 |  | Required for RNA editing in plant mitochondria. | Mitochondrion: 1.0 MitoProt2. | NR | NR | [81–83] |
| COD1 | AT2G35030 | O64766 |  | PLS-subfamily PPR protein which is involved in the editing of two distant sites in the *COX2* transcript. | FP [21] | NR | NR | [84] |
| MEF13 | AT3G02330 | Q9FWA6 |  | Involved in C-to-U editing of mitochondrial RNA. Required for RNA editing at 8 sites in 6 different mRNAs in mitochondria. | FP [21]  MS [79] | NR | MORF1/RIP8, MORF8/RIP1 [79] | [79] |
| ORRM4 | AT1G74230 | Q9C909 | Organelle RNA Recognition Motif-containing protein. | Functions as major mitochondrial editing factor. Controls 44% of the mitochondrial editing sites. | FP [74,85]  MS [4,6,9,37,86] | NR | MORF8/RIP1 RBG3/ORRM3 [74] | [74,85,87] |
| mCSF-1 | AT4G31010 | Q8VYD9 | CRS2-associated factor 1, mitochondrial. | CRM family member required for the processing of many mitochondrial introns. Involved in the biogenesis of respiratory complexes I and IV. | Mitochondrion: 1.0 [21] | NR | NR | [88] |
| NMAT2 | AT5G46920 | Q9FJR9 | Nuclear intron maturase 2. | Nuclear-encoded maturase required for splicing of group-II introns in mitochondria. Associated to a large ribonucleoprotein complex in mitochondria containing group-II intron RNAs. | FP [89] | NR | NR | [89–91] |
| PMH2 | AT3G22330 | Q9LUW5 | Putative mitochondrial RNA helicase 2. | Mitochondria-located protein required for efficient group II intron splicing in mitochondria. | FP [11]  MS [4,6,9,10] | NR | NR | [11,92] |
| MIO24.13 | AT5G51740 | Q9FLI5 | Peptidase family M48 family protein. | Peptidase family M48 family protein. | MS [4,6–10] | NR | NR | NR |
| Mitochondrial glycoprotein family | AT5G02050 | Q9LZM6 | Mitochondrial glycoprotein family protein. |  | MS [7,9] | NR | AT4G25200 [11] | NR |
|  | AT1G80720 | Q9SAI6 |  | Mitochondrial glycoprotein family protein. | MS [9] | NR | NR | NR |
|  | AT1G15870 | Q9LMP8 |  |  | Mitochondrion: 1.0 [21] | NR | NR | NR |
|  | AT3G55605 | Q8LCT2 |  |  | MS [9,87] | NR | AT5G47590 [11] | NR |
|  | AT5G05990 | Q9FI87 |  |  | MS [6,9] | P-1 [93] | NR | NR |
|  | AT2G39795 | Q8W487 |  |  | MS [4,6,7,87]  TP: 1-52 [26] | UBI-100  P-1 [94,95] | NR | [10,11] |
|  | AT4G31930 | Q9AST4 |  |  | MS [9,37] | NR | NR | NR |
|  | AT2G41600 | Q3E7H1 |  |  | MS [9] | UBI-7 [19] | NR | NR |
| mtHSC70-1 | AT4G37910 | Q8GUM2 | Heat shock 70 kDa protein 9. | Stabilize preexistent proteins against aggregation and mediate the folding of newly translated polypeptides. | MS [11]  TP: 1-46 [26] | NR  P [12,95,96, ] | AT5G09230 [97] | [98] |
| mtHSC70-2 | AT5G09590 | Q9LDZ0 | Heat shock 70 kDa protein 10. |  | MS [11]  TP: 1-50 [26] | P [95] | AT4G11260 [98] | [98,99] |
| MATR | ATMG00520 | P93307 | Maturase | Intron maturase, type II family protein. | Mitochondrion: 1.0 [21] | P-5 [100–102] | NR | NR |

^a^ Experimental evidence for mitochondrial localization and strategy of identification: FP (Fusion to Fluorescence Proteins), MS (Mass Spectrometry). ^b^ PTMS: Posttranslational protein modifications. The information was taken from the PLMD3.0 (http://plmd.biocuckoo.org/index.php, [96]) and PhosPhAT 4.0 (http://phosphat.uni-hohenheim.de/phosphat.html, [102]) databases. UBI: Ubiquitiation, Ace: Acetylation, P: Phosphorylation. ^c^ PPI: Reported evidences about Protein-Protein Interactions. Experimental information about PPI is from the UniProtKB database (http://www.uniprot.org/help/evidences, [26]). ^d^ Experimental evidence about the respective COX-related protein. NR: Not Reported; TP: presence of canonical Transit Peptide; DIS: Disulfide bridge.

**References**

1. Millar, A.; Eubel, H.; Jänsch, L.; Kruft, V.; Heazlewood, J.; Braun, H. Mitochondrial cytochrome *c* oxidase and succinate dehydrogenase complexes contain plant specific subunits. *Plant Mol. Biol*. **2004**, *56*, 77–90, doi:10.1007/s11103-004-2316-2.
2. Unseld, M.; Marienfeld, J.; Brandt, P.; Brennicke, A. The mitochondrial genome of *Arabidopsis thaliana* contains 57 genes in 366,924 nucleotides. *Nat. Genet*. **1997**, *15*, 57–61, doi:10.1038/ng0197-57.
3. Eubel, H.; Jansch, L.; Braun, H.P. New Insights into the Respiratory Chain of Plant Mitochondria. Supercomplexes and a Unique Composition of Complex II. *Plant Physiol.* **2003**, *133*, 274–286, doi:10.1104%2Fpp.103.024620.
4. Taylor, N.; Heazlewood, J.; Millar, A. The *Arabidopsis thaliana* 2-D gel mitochondrial proteome: Refining the value of reference maps for assessing protein abundance, contaminants and post-translational modifications. *Proteomics* **2011**, *11*, 1720–1733, doi:10.1002/pmic.201000620.
5. Duncan, O.; Taylor, N.; Carrie ,C.; Eubel, H.; Kubiszewski-Jakubiak, S.; Zhang, B.; Narsai, R.; Millar, A.; Whelan, J. Multiple Lines of Evidence Localize Signaling, Morphology, and Lipid Biosynthesis Machinery to the Mitochondrial Outer Membrane of *Arabidopsis*. *Plant Physiol*. **2011**, *157*, 1093–1113, doi:10.1104/pp.111.183160.
6. Klodmann, J.; Senkler, M.; Rode, C.; Braun, H. Defining the Protein Complex Proteome of Plant Mitochondria. *Plant Physiol*. **2011**, *157*, 587–598, doi:10.1104/pp.111.182352.
7. Nikolovski, N.; Rubtsov, D.; Segura, M.P.; Miles, G.P.; Stevens, T.J.; Dunkley, T.P.J.; Munro, S.; Lilley, K.S.; Dupree, P. Putative glycosyltransferases and other plant Golgi apparatus proteins are revealed by LOPIT proteomics. *Plant Physiol*. **2012**, *160*, 1037–1051, doi:10.1104/pp.112.204263.
8. Fromm, S.; Senkler, J.; Eubel, H.; Peterhänsel, C.; Braun, H. Life without complex I: Proteome analyses of an *Arabidopsis* mutant lacking the mitochondrial NADH dehydrogenase complex. *J. Exp. Bot*. **2016**, *67*, 3079–3093, doi:10.1093/jxb/erw165.
9. Senkler, J.; Senkler, M.; Eubel, H.; Hildebrandt, T.; Lengwenus, C.; Schertl, P.; Schwarzländer, M.; Wagner, S.; Wittig, I.; Braun, H.P. The mitochondrial complexome of *Arabidopsis thaliana*. *Plant J*. **2017**, *89*, 1079–1092, doi:10.1111/tpj.13448.
10. Heazlewood, J.L.; Tonti-Filippini, J.S.; Gout, A.M.; Day, D.A.; Whelan, J.; Millar, A.H. Experimental analysis of the *Arabidopsis* mitochondrial proteome highlights signaling and regulatory components, provides assessment of targeting prediction programs, and indicates plant-specific mitochondrial proteins. *Plant Cell* **2004**, *16*, 241–256, doi:10.1105/tpc.016055.
11. Carrie, C.; Venne, A.; Zahedi, R.; Soll, J. Identification of cleavage sites and substrate proteins for two mitochondrial intermediate peptidases in *Arabidopsis thaliana*. *J. Exp. Bot*. **2015**, *66*, 2691–2708, doi:10.1093/jxb/erv064.
12. Roitinger, E.; Hofer, M.; Köcher, T.; Pichler, P.; Novatchkova, M.; Yang, J.; Schlögelhofer, P.; Mechtler, K. Quantitative Phosphoproteomics of the ATaxia Telangiectasia-Mutated (ATM) and ATaxia Telangiectasia-Mutated and Rad3-related (ATR) Dependent DNA Damage Response in *Arabidopsis thaliana*. *Mol. Cell. Proteom.* **2015**, *14*, 556–557, doi:10.1074%2Fmcp.M114.040352.
13. Wang, X.; Bian, Y.; Cheng, K.; Gu, L.F.; Ye, M.; Zou, H.; Sun, S.S.; He, J. A large-scale protein phosphorylation analysis reveals novel phosphorylation motifs and phosphoregulatory networks in *Arabidopsis*. *J. Proteom.* **2013**, *78*, 486–498, doi:10.1016/j.jprot.2012.10.018.
14. Braun, P.; Arabidopsis Interactome Mapping Consortium. All Evidence for Network Evolution in an *Arabidopsis* Interactome Map. *Science* **2011**, *333*, 601–607, doi:10.1126/science.1203877.
15. Welchen, E.; Chan, R.L.; Gonzalez, D.H. Metabolic regulation of genes encoding cytochrome *c* and cytochrome *c* oxidase subunit Vb in *Arabidopsis*. *Plant Cell Environ*. **2002**, *25*, 1605–1615, doi:10.1046/j.1365-3040.2002.00940.x.
16. Welchen, E.; Chan, R.L.; Gonzalez, D.H. The promoter of the *Arabidopsis* nuclear gene COX5b-1, encoding subunit 5b of the mitochondrial cytochrome *c* oxidase, directs tissue-specific expression by a combination of positive and negative regulatory elements. *J. Exp. Bot*. **2004**, *55*, 1997–2004, doi:10.1093/jxb/erh223.
17. Comelli, R.N.; Viola, I.L.; Gonzalez, D.H. Characterization of promoter elements required for expression and induction by sucrose of the *Arabidopsis COX5b-1* nuclear gene, encoding the zinc-binding subunit of cytochrome c oxidase. *Plant Mol. Biol*. **2009**, *69*, 729–743, doi:10.1007/s11103-008-9451-0.
18. Comelli, R.N.; Welchen, E.; Kim, H.J.; Hong, J.C.; Gonzalez, D.H. Delta subclass HD-Zip proteins and a B-3 AP2/ERF transcription factor interact with promoter elements required for expression of the *Arabidopsis* cytochrome c oxidase 5b-1 gene. *Plant Mol. Biol*. **2012**, *80*, 157–167, doi:10.1007/s11103-012-9935-9.
19. Walton, A.; Stes, E.; Cybulski, N.; Van Bel, M.; Iñigo, S.; Durand, A.; Timmerman, E.; Heyman, J.; Pauwels, L.; De Veylder, L.; et al. It’s Time for Some “Site”-Seeing: Novel Tools to Monitor the Ubiquitin Landscape in *Arabidopsis thaliana*. *Plant Cell* **2016**, *28*, 6–16, doi:10.1105/tpc.15.00878.
20. Comelli, R.N.; Gonzalez, D.H. Identification of regulatory elements involved in expression and induction by sucrose and UV-B light of the *Arabidopsis thaliana* *COX5b-2* gene, encoding an isoform of cytochrome *c* oxidase subunit 5b. *Physiol. Plant.* **2009**, *137*, 213–224, doi:10.1111/j.1399-3054.2009.01285.x.
21. Hooper, C.M.; Castleden, I.R.; Tanz, S.K.; Aryamanesh, N.; Millar, A.H. SUBA4: The interactive data analysis centre for *Arabidopsis* subcellular protein locations. *Nucleic Acids Res*. **2017**, *45*, D1064–D1074, doi:10.1093/nar/gkw1041.
22. Nakagawa, T.; Maeshima, M.; Nakamura, K.; Asahi, T. Molecular cloning of a cDNA for the smallest nuclear-encoded subunit of sweet potato cytochrome *c* oxidase. Analysis with the cDNA of the structure and import into mitochondria of the subunit. *Eur. J. Biochem*. **1990**, *191*, 557–561, doi:10.1111/j.1432-1033.1990.tb19157.x
23. Curi, G.C.; Chan, R.L.; Gonzalez, D.H. Genes encoding cytochrome *c* oxidase subunit 5c from sunflower (*Helianthus annuus* L.) are regulated by nitrate and oxygen availability. *Plant Sci*. **2002**, *163*, 897–905, doi:10.1016/S0168-9452(02)00237-6.
24. Curi, G.C.; Chan, R.L.; Gonzalez, D.H. The leader intron of *Arabidopsis thaliana* genes encoding cytochrome c oxidase subunit 5c promotes high-level expression by increasing transcript abundance and translation efficiency. *J. Exp. Bot*. **2005**, *56*, 2563–2571, doi:10.1093/jxb/eri250.
25. Cutler, S.; Ehrhardt, D.; Griffitts, J.; Somerville, C. Random GFP::cDNA fusions enable visualization of subcellular structures in cells of *Arabidopsis* at a high frequency. *Proc. Natl. Acad. Sci. USA* **2000**, *97*, 3718–3723, doi:10.1073/pnas.97.7.3718.
26. Wu, C.H.; Apweiler, R.; Bairoch, A.; Natale, D.A.; Barker, W.C.; Boeckmann, B.; Ferro, S.; Gasteiger, E.; Huang, H.; Lopez, R.; et al. The Universal Protein Resource (UniProt): An expanding universe of protein information. *Nucleic Acids Res*. **2006**, *34*, D187–D191, doi:10.1093/nar/gkj161.
27. Curi, G.C.; Welchen, E.; Chan, R.L.; Gonzalez, D.H. Nuclear and mitochondrial genes encoding cytochrome *c* oxidase subunits respond differently to the same metabolic factors. *Plant Physiol.* *Biochem*. **2003**, *41*, 689–693, doi:[10.1016/S0981-9428(03)00093-7](https://doi.org/10.1016/S0981-9428(03)00093-7).
28. König, A.; Hartl, M.; Boersema, P.; Mann, M.; Finkemeier, I. The mitochondrial lysine acetylome of *Arabidopsis*. *Mitochondrion* **2014**, *19*, 252–260, doi:10.1016/j.mito.2014.03.004.
29. Bienvenut, W.; Sumpton, D.; Martinez, A.; Lilla, S.; Espagne, C.; Meinnel, T.; Giglione, C. Comparative large scale characterization of plant versus mammal proteins reveals similar and idiosyncratic *N*-α-acetylation features. *Mol. Cell. Proteom.* **2012**, *11*, doi:10.1074/mcp.
30. Dortay, H.; Gruhn, N.; Pfeifer, A.; Schwerdtner, M.; Schmulling, T.; Heyl, A. Toward an interaction map of the two-component signaling pathway of *Arabidopsis thaliana*. *J. Proteome Res*. **2008**, *7*, 3649–3660, doi:10.1021/pr0703831.
31. Ohtsu, K.; Nakazono, M.; Tsutsumi, N.; Hirai, A. Characterization and expression of the genes for cytochrome *c* oxidase subunit VIb (COX6b) from rice and *Arabidopsis thaliana*. *Gene* **2001**, *264*, 233–239, doi:10.1016/S0378-1119(01)00334-1.
32. Saish, D.; Nakazono, M.; Lee, K.; Tsutsumi, N.; Akita, S.; Hirai, A. The gene for alternative oxidase-2 (AOX2) from *Arabidopsis thaliana* consists of five exons unlike other AOX genes and is transcribed at an early stage during germination. *Genes Genet. Syst.* **2001**, *76*, 89–97, doi:doi 10.1266/ggs.76.89.
33. Mufarrege, E.F.; Curi, G.C.; Gonzalez, D.H. Common sets of promoter elements determine the expression characteristics of three *Arabidopsis* genes encoding isoforms of mitochondrial cytochrome c oxidase subunit 6b. *Plant Cell Physiol*. **2009**, *50*, 1393–1399, doi:10.1093/pcp/pcp080.
34. Uno, Y.; Rodriguez Milla, M.A.; Maher, E.; Cushman, J. Identification of proteins that interact with catalytically active calcium-dependent protein kinases from *Arabidopsis*. *Mol. Genet. Genom.* **2009**, *281*, 375–390, doi:10.1007/s00438-008-0419-1.
35. Hwang, S.T.; Li, H.; Alavilli, H.; Lee, B.H.; Choi, D. Molecular and physiological characterization of *ATHIGD1* in Arabidopsis. *Biochem. Biophys. Res. Comm*un. **2017**, *487*, 881–886, doi:10.1016/j.bbrc.2017.04.146.
36. Bürkle, L.; Meyer, S.; Dortay, H.; Lehrach, H.; Heyl, A. In vitro recombination cloning of entire cDNA libraries in *Arabidopsis thaliana* and its application to the yeast two-hybrid system. *Funct. Int. Genom*. **2005**, *5*, 175–183, doi:10.1007/s10142-005-0134-5.
37. Lee, C.; Eubel, H.; O’Toole, N.; Millar, A. Combining proteomics of root and shoot mitochondria and transcript analysis to define constitutive and variable components in plant mitochondria. *Phytochemistry* **2011**, *72*, 1092–1098, doi:10.1016/j.phytochem.2010.12.004.
38. Sakamoto, W.; Spielewoy, N.; Bonnard, G.; Murata, M.; Wintz, H. Mitochondrial localization of ATOXA1, an *Arabidopsis* homologue of yeast Oxa1p involved in the insertion and assembly of protein complexes in mitochondrial inner membrane. *Plant Cell Physiol*. **2000**, *41*, 1157–1163, doi:10.1093/pcp/pcd045.
39. Hamel, P.; Sakamoto, W.; Wintz, H.; Dujardin, G. Functional complementation of an oxa1- yeast mutation identifies an *Arabidopsis* *thaliana* cDNA involved in the assembly of respiratory complexes. *Plant J*. **1997**, *6*, 1319–1327, doi:10.1046/j.1365-313x.1997.12061319.x.
40. [Mansilla, N](https://www.ncbi.nlm.nih.gov/pubmed/?term=Mansilla%20N%5BAuthor%5D&cauthor=true&cauthor_uid=26246612).; [Garcia, L](https://www.ncbi.nlm.nih.gov/pubmed/?term=Garcia%20L%5BAuthor%5D&cauthor=true&cauthor_uid=26246612).; [Gonzalez, D.H](https://www.ncbi.nlm.nih.gov/pubmed/?term=Gonzalez%20DH%5BAuthor%5D&cauthor=true&cauthor_uid=26246612).; [Welchen, E](https://www.ncbi.nlm.nih.gov/pubmed/?term=Welchen%20E%5BAuthor%5D&cauthor=true&cauthor_uid=26246612). ATCOX10, a protein involved in haem *o* synthesis during cytochrome *c* oxidase biogenesis, is essential for plant embryogenesis and modulates the progression of senescence*. J. Exp. Bot*. **2014**, *66*, 6761–6775, doi:10.1093/jxb/erv381.
41. Takubo, K.; Morikawa, T.; Nonaka, Y.; Mizutani, M.; Takenaka, S.; Takabe, K.; Takahashi, M.; Ohta, D. Identification and molecular characterization of mitochondrial ferredoxins and ferredoxin reductase from *Arabidopsis*. *Plant Mol. Biol*. **2003**, *52*, 817–830, doi:10.1023/A:102501581.
42. Picciocchi, A.; Douce, R.; Alban, C. The plant biotin synthase reaction. Identification and characterization of essential mitochondrial accessory protein components. *J. Biol. Chem*. **2003**, *278*, 24966–24975, doi:10.1074/jbc.M302154200.
43. Van Leene, J.; Hollunder, J.; Eeckhout, D.; Persiau, G.; Van De Slijke, E.; Stals, H.; Van Isterdael, G.; Verkest, A.; Neirynck.; Buffel, Y.; et al. Targeted interactomics reveals a complex core cell cycle machinery in *Arabidopsis thaliana*. *Mol. Syst. Biol.* **2010**, *6*, 397, doi:10.1038/msb.2010.53.
44. Steinebrunner, I.; Landschreiber, M.; Krause-Buchholz, U.; Teichmann, J.; Rödel, G. HCC1, the Arabidopsis homologue of the yeast mitochondrial copper chaperone SCO1, is essential for embryonic development. *J. Exp. Bot*. **2011**, *62*, 319–330, doi:10.1093/jxb/erq269.
45. Attallah, C.V.; Welchen, E.; Martin, A.P.; Spinelli, S.V.; Bonnard, G.; Palatnik, J.F.; Gonzalez, D.H. Plants contain two SCO proteins that are differentially involved in cytochrome *c* oxidase function and copper and redox homeostasis. *J. Exp. Bot*. **2011**, *62*, 4281–4294, doi:10.1093/jxb/err138.
46. Steinebrunner, I.; Gey, U.; Andres, M.; Garcia, L.; Gonzalez, D.H. Divergent functions of the Arabidopsis mitochondrial SCO proteins: HCC1 is essential for COX activity while HCC2 is involved in the UV-B stress response. *Front. Plant Sci*. **2014**, *5*, 87, doi:10.3389/fpls.2014.00087.eCollection2014.
47. Radin, I.; Mansilla, N.; Rödel, G.; Steinebrunner, I. The *Arabidopsis* COX11 Homolog is Essential for Cytochrome *c* Oxidase Activity. *Front. Plant Sci*. **2015**, *6*, 1091, doi:10.3389/fpls.2015.01091.
48. Garcia, L.; Welchen, E.; Gey, U.; Arce, A.L.; Steinebrunner, I.; Gonzalez, D.H. The cytochrome *c* oxidase biogenesis factor ATCOX17 modulates stress responses in *Arabidopsis*. *Plant Cell Environ*. **2016**, *39*, 628–644, doi:10.1111/pce.12647.
49. Zhang, H.; Zhou, H.; Berke, L.; Heck, A.J.; Mohammed, S.; Scheres, B.; Menke, F.L. Quantitative phosphoproteomics after auxin-stimulated lateral root induction identifies an SNX1 protein phosphorylation site required for growth. *Mol. Cell. Proteom.* **2013**, *12*, 1158–1169, doi:10.1074/mcp.M112.021220.
50. Balandin, T.; Castresana, C. ATCOX17, an Arabidopsis homolog of the yeast copper chaperone COX17. *Plant Physiol*. **2002**, *129*, 1852–1857, doi:10.1104/pp.010963.
51. Wintz, H.; Vulpe, C. Plant copper chaperones. *Biochem. Soc. Trans*. **2002**, *30*, 732–735, doi:10.1042/BST0300732.
52. Attallah, C.V.; Welchen, E.; Gonzalez, D.H. The promoters of *Arabidopsis thaliana* genes ATCOX17-1 and -2, encoding a copper chaperone involved in cytochrome *c* oxidase biogenesis, are preferentially active in roots and anthers and induced by biotic and abiotic stress. *Physiol. Plant.* **2007**, *129*, 123–134, doi:10.1111/j.1399-3054.2006.00776.x.
53. Attallah, C.V.; Welchen, E.; Pujol, C.; Bonnard, G.; Gonzalez, D.H. Characterization of *Arabidopsis thaliana* genes encoding functional homologues of the yeast metal chaperone Cox19p, involved in cytochrome *c* oxidase biogenesis. *Plant Mol. Biol*. **2007**, *65*, 343–355, doi:10.1007/s11103-007-9224-1.
54. Caillaud, M.C.; Wirthmueller, L.; Sklenar, J.; Findlay, K.; Piquerez, S.J.; Jones, A.M.; Robatzek, S.; Jones, J.D.; Faulkner, C. The plasmodesmal protein PDLP1 localises to haustoria-associated membranes during downy mildew infection and regulates callose deposition. *PLoS Pathog*. **2014**, *10*, e1004496, doi:10.1371/journal.ppat.1004496.
55. Ueoka-Nakanishi, H.; Sazuka, T.; Nakanishi, Y.; Maeshima, M.; Mori, H.; Hisabori, T. Thioredoxin *h* regulates calcium dependent protein kinases in plasma membranes. *FEBS J*. **2013**, *280*, 3220–3231, doi:10.1111/febs.12301.
56. Takabatake, R.; Hata, S.; Taniguchi, M.; Kouchi, H.; Sugiyama, T.; Izui, K. Isolation and characterization of cDNAs encoding mitochondrial phosphate transporters in soybean, maize, rice, and Arabidopis. *Plant Mol. Biol*. **1999**, *40*, 479–486, doi:10.1023/A:100628500.
57. Picault, N.; Hodges, M.; Palmieri, L.; Palmieri, F. The growing family of mitochondrial carriers in *Arabidopsis*. *Trends Plant Sci*. **2004**, *9*, 138–146, doi:10.1016/j.tplants.2004.01.007.
58. Zhu, W.; Miao, Q.; Sun, D.; Yang, G.; Wu, C.; Huang, J.; Zheng, C. The mitochondrial phosphate transporters modulate plant responses to salt stress via affecting ATP and gibberellin metabolism in *Arabidopsis thaliana*. *PLoS ONE* **2012**, *7*, e43530, doi:10.1371/journal.pone.0043530.
59. Van Aken, O.; Zhang, B.; Carrie, C.; Uggalla, V.; Paynter, E.; Giraud, E.; Whelan. J. Defining the mitochondrial stress response in *Arabidopsis thaliana*. *Mol. Plant* **2009**, *2*, 1310–1324, doi:10.1093/mp/ssp053.
60. Tarantino, D.; Morandini, P.; Ramirez, L.; Soave, C.; Murgia, I. Identification of an *Arabidopsis* mitoferrinlike carrier protein involved in Fe metabolism. *Plant Physiol. Biochem*. **2011**, *49*, 520–529, doi:10.1016/j.plaphy.2011.02.003.
61. Finkemeier, I.; Laxa, M.; Miguet, L.; Howden, A.; Sweetlove, L. Proteins of Diverse Function and Subcellular Location Are Lysine Acetylated in *Arabidopsis*. *Plant Physiol*. **2011**, *155*, 1779–1790, doi:10.1104/pp.110.171595.
62. Kosarev, P.; Mayer, K.; Hardtke, C. Evaluation and classification of RING-finger domains encoded by the *Arabidopsis* genome. *Genome Biol.* **2002**, *3*, doi:10.1186/gb-2002-3-4-research0016
63. Lurin, C.; Andres, C.; Aubourg, S.; Bellaoui, M.; Bitton, F.; Bruyère, C.; Caboche, M.; Debast, C.; Gualberto, J.; Hoffmann, B.; et al. Genome-wide analysis of *Arabidopsis* pentatricopeptide repeat proteins reveals their essential role in organelle biogenesis. *Plant Cell* **2004**, *16*, 2089–2103, doi:10.1105/tpc.104.022236.
64. Jonietz, C.; Forner, J.; Holzle, A.; Thuss, S.; Binder, S. RNA PROCESSING FACTOR2 is required for 5′ end processing of nad9 and cox3 mRNAs in Mitochondria of *Arabidopsis thaliana*. *Plant Cell* **2010**, *22*, 443–453, doi:10.1105/tpc.109.066944.
65. Narsai, R.; Law, S.R.; Carrie, C.; Xu, L.; Whelan, J. In-depth temporal transcriptome profiling reveals a crucial developmental switch with roles for RNA processing and organelle metabolism that are essential for germination in *Arabidopsis*. *Plant Physiol*. **2011**, *157*, 1342–1362, doi:10.1104/pp.111.183129.
66. Colas des Francs-Small, C.; Kroeger, T.; Zmudjak, M.; Ostersetzer-Biran, O.; Rahimi, N.; Small, I.; Barkan, A. A PORR domain protein required for *rpl2* and *ccmFC* intron splicing and for the biogenesis of *c*-type cytochromes in *Arabidopsis* mitochondria. *Plant J*. **2012**, *69*, 996–1005, doi:10.1111/j.1365-313X.2011.04849.x.
67. Bentolila, S.; Hellera, W.P.; Suna, T.; Babinaa, A.M.; Frisob, G.; Van Wijkb, K.J.; Hanson, M.R. RIP1, a member of an *Arabidopsis* protein family, interacts with the protein RARE1 and broadly affects RNA editing. *Proc. Natl. Acad. Sci. USA* **2012**, *109*, E1453–E1461, doi:10.1073/pnas.1121465109.
68. Zehrmann, A.; Haertel, B.; Glass, F.; Bayer-Csaszar, E.; Obata, T.; Meyer, E.; Brennicke, A.; Takenaka, M. Selective homo- and heteromer interactions between the multiple organellar RNA editing factor (MORF) proteins in *Arabidopsis* *thaliana*. *J. Biol. Chem*. **2015**, *290*, 6445–6456, doi:10.1074/jbc.M114.602086.
69. Barrôco, R.M.; De Veylder, L.; Magyar, Z.; Engler, G.; Inzé, D.; Mironov, V. Novel complexes of cyclin-dependent kinases and a cyclin-like protein from *Arabidopsis* *thaliana* with a function unrelated to cell division. *Cell. Mol. Life Sci*. **2003**, *60*, 401–412, doi:10.1007/s000180300033.
70. Bentolila, S.; Oh, J.; Hanson, M.R.; Bukowski, R. Comprehensive high-resolution analysis of the role of an *Arabidopsis* gene family in RNA editing. *PLoS Genet*. **2013**, *9*, e1003584, doi:10.1371/journal.pgen.1003584.
71. Haertel, B.; Zehrmann, A.; Verbitskiy, D.; van der Merwe, J.A.; Brennicke, A.; Takenaka, M. MEF10 is required for RNA editing at *nad2*-842 in mitochondria of *Arabidopsis thaliana* and interacts with MORF8. *Plant Mol. Biol*. **2013**, *81*, 337–346, doi:10.1007/s11103-012-0003-2.
72. Zhang, F.; Tang, W.; Hedtke, B.; Zhong, L.; Liu, L.; Peng, L.; Lu, C.; Grimm, B.; Lin, R. Tetrapyrrole biosynthetic enzyme protoporphyrinogen IX oxidase 1 is required for plastid RNA editing. *Proc. Natl. Acad. Sci. USA* **2014**, *111*, 2023–2028, doi:10.1073/pnas.1316183111.
73. Zhang, H.D.; Cui, Y.L.; Huang, C.; Yin, Q.Q.; Qin, X.M.; Xu, T.; He, X.F.; Zhang, Y.; Li, Z.R.; Yang, Z.N. PPR protein PDM1/SEL1 is involved in RNA editing and splicing of plastid genes in *Arabidopsis* *thaliana*. *Photosyn. Res*. **2015**, *126*, 311–321, doi:10.1007/s11120-015-0171-4.
74. Shi, X.; Germain, A.; Hanson, M.R.; Bentolila, S. RNA Recognition Motif-Containing Protein ORRM4 Broadly Affects Mitochondrial RNA Editing and Impacts Plant Development and Flowering. *Plant Physiol*. **2016**, *170*, 294–309, doi:10.1104/pp.15.01280.
75. Sun, T.; Shi, X.; Friso, G.; Van Wijk, K.; Bentolila, S.; Hanson, M.R. A zinc finger motif-containing protein is essential for chloroplast RNA editing. *PLoS Genet.* **2015**, *13*, e1005028, doi:10.1371/journal.pgen.1005028.
76. Brehme, N.; Bayer-Csaszar, E.; Glass, F.; Takenaka, M. The DYW subgroup PPR protein MEF35 targets RNA editing sites in the mitochondrial rpl16, nad4 and cob mRNAs in *Arabidopsis* *thaliana*. *PLoS ONE* **2015**, *10*, e0140680, doi:10.1371/journal.pone.0140680.
77. Hackett, J.B.; Shi, X.; Kobylarz, A.T.; Lucas, M.K.; Wessendorf, R.L.; Hines, K.M.; Bentolila, S.; Hanson, M.R.; Lu, Y. An organelle RNA recognition motif protein is required for photosynthetic subunit psbF transcript editing. *Plant Physiol*. **2017**, *173*, 2278–2293, doi:10.1104/pp.16.01623.
78. Glass, F.; Hartel, B.; Zehrmann, A.; Verbitskiy, D.; Takenaka, M. MEF13 Requires MORF3 and MORF8 for RNA Editing at Eight Targets in Mitochondrial mRNAs in *Arabidopsis thaliana*. *Mol. Plant* **2015**, *8*, 1466-1477, doi:10.1016/j.molp.2015.05.008.
79. Takenaka, M.; Zehrmann, A.; Verbitskiy, D.; Kugelmann, M.; Härtel, B.; Brennicke, A. Multiple organellar RNA editing factor (MORF) family proteins are required for RNA editing in mitochondria and plastids of plants. *Proc. Natl. Acad. Sci. USA* **2012**, *109*, 5104–5109, doi:10.1073/pnas.1202452109.
80. Takenaka, M.; Verbitskiy, D.; Zehrmann, A.; Brennicke, A. Reverse genetic screening identifies five E-class PPR proteins involved in RNA editing in mitochondria of *Arabidopsis* *thaliana*. *J. Biol. Chem*. **2010**, *285*, 27122–27129, doi:10.1074/jbc.M110.128611.
81. Verbitskiy, D.; Zehrmann, A.; Van Der Merwe, J.; Brennicke, A.; Takenaka, M. The PPR protein encoded by the lovastatin insensitive 1 gene is involved in RNA editing at three sites in mitochondria of *Arabidopsis thaliana.* *Plant J*. **2010**, *61*, 446–455, doi:10.1111/j.1365-313X.2009.04076.x.
82. Kobayashi, K.; Suzuki, M.; Tang, J.; Nagata, N.; Ohyama, K.; Seki, H.; Kiuchi, R.; Kaneko, Y.; Nakazawa, M.; Matsui, M.; et al. Lovastatin insensitive 1, a Novel pentatricopeptide repeat protein, is a potential regulatory factor of isoprenoid biosynthesis in *Arabidopsis*. *Plant Cell Physiol.* **2007**, *48*, 322–331, doi:10.1093/pcp/pcm005.
83. Arenas-M, A.; Zehrmann, A.; Moreno, S.; Takenaka, M.; Jordana, X. The pentatricopeptide repeat protein MEF26 participates in RNA editing in mitochondrial cox3 and nad4 transcripts. *Mitochondrion* **2014**, *19*, 126–134, doi:10.1016/j.mito.2014.08.006.
84. Dahan, J.; Tcherkez, G.; Macherel, D.; Benamar, A.; Belkram, K.; Quadrado, M.; Arnal, N.; Mireau, H. Disruption of the *CYTOCHROME C OXIDASE DEFICIENT1* gene leads to cytochrome *c* oxidase depletion and reorchestrated respiratory metabolism in *Arabidopsis*. *Plant Physiol*. **2014**, *166*, 1788–1802, doi:10.1104/pp.114.248526.
85. Vermel, M.; Guermann, B.; Delage, L.; Grienenberger, J.M.; Maréchal-Drouard, L.; Gualberto, J.M. A family of RRM-type RNA-binding proteins specific to plant mitochondria. *Proc. Natl. Acad. Sci. USA* **2002**, *99*, 5866–5871, doi:10.1073%2Fpnas.092019599.
86. Ito, J.; Heazlewood, J.L.; Millar, A.H. Analysis of the soluble ATP-binding proteome of plant mitochondria identifies new proteins and nucleotide triphosphate interactions within the matrix. *J. Proteome* **2006**, *5*, 3459–3469, doi:10.1021/pr060403j.
87. Mangeon, A.; Junqueira, R.M.; Sachetto-Martins, G. Functional diversity of the plant glycine-rich proteins superfamily. *Plant Signal. Behav*. **2010**, *5*, 99–104, doi:10.4161/psb.5.2.10336.
88. Zmudjak, M.; Colas des Francs-Small, C.; Keren, I.; Shaya, F.; Belausov, E.; Small, I.; Ostersetzer-Biran, O. mCSF1, a nucleus-encoded CRM protein required for the processing of many mitochondrial introns, is involved in the biogenesis of respiratory complexes I and IV in *Arabidopsis*. *New Phytol.* **2013**, *199*, 379–394, doi:10.1111/nph.12282.
89. Keren, I.; Bezawork-Geleta, A.; Kolton, M.; Maayan, I.; Belausov, E.; Levy, M.; Mett, A.; Gidoni, D.; Shaya, F.; Ostersetzer-Biran, O. ATnMat2, a nuclear-encoded maturase required for splicing of group-II introns in *Arabidopsis* mitochondria. *RNA* **2009**, *15*, 2299–2311, doi:10.1261/rna.1776409.
90. Keren, I.; Tal, L.; des Francs-Small, C.C.; Araujo, W.L.; Shevtsov, S.; Shaya, F.; Fernie, A.R.; Small, I.; Ostersetzer-Biran, O. nMAT1, a nuclear-encoded maturase involved in the trans-splicing of nad1 intron 1, is essential for mitochondrial complex I assembly and function. *Plant J*. **2012**, *71*, 413–426, doi:10.1111/j.1365-313X.2012.04998.x.
91. Brown, G.G.; Colas des Francs-Small, C.; Ostersetzer-Biran, O. Group II intron splicing factors in plant mitochondria. *Front. Plant Sci.* **2014**, *5*, 35, doi:10.3389/fpls.2014.00035.
92. Zmudjak, M.; Shevtsov, S.; Sultan, L.D.; Keren, I. Analysis of the Roles of the Arabidopsis nMAT2 and PMH2 Proteins Provided with New Insights into the Regulation of Group II Intron Splicing in Land-Plant Mitochondria. *Int. J. Mol. Sci*. **2017**, *18*, 2428, doi:10.3390/ijms18112428.
93. Engelsberger, W.R.; Schulze, W.X. Nitrate and ammonium lead to distinct global dynamic phosphorylation patterns when resupplied to nitrogen-starved Arabidopsis seedlings. *Plant J*. **2012**, *69*, 978–995, doi:10.1111/j.1365-313X.2011.04848.
94. Ito, J.; Taylor, N.; Castleden, I.; Weckwerth, W.; Millar, A.; Heazlewood, J. A survey of the *Arabidopsis thaliana* mitochondrial phosphoproteome. *Proteomics* **2009**, *9*, 4229–4240, doi:10.1002/pmic.200900064.
95. Nakagami, H.; Sugiyama, N.; Mochida, K.; Daudi, A.; Yoshida, Y.; Toyoda, T.; Tomita, M.; Ishihama, Y.; Shirasu, K. Large-Scale Comparative Phosphoproteomics Identifies Conserved Phosphorylation Sites in Plants. *Plant Physiol*. **2010**, *153*, 1161–1174, doi:10.1104/pp.110.157347.
96. Xu, H.; Zhou, J.; Lin, S.; Deng, W.; Zhang, Y.; Xue, Y. PLMD: An updated data resource of protein lysine modifications. *J. Genet. Genom.* **2017**, *44*, 243–250, doi:10.1093%2Fnar%2Fgkw1041.
97. Koenig, A.C. Hartl, M.; Pham, P.A.; Laxa, M.; Boersema, P.J.; Orwat, A.; Kalitventseva, I.; Ploechinger, M.; Braun, H.P.; Leister, D.; et al. The Arabidopsis class II sirtuin is a lysine deacetylase and interacts with mitochondrial energy metabolism. *Plant Physiol*. **2014**, *164*, 1401–1414, doi:10.1104/pp.113.232496.
98. Noel, L.D.; Cagna, G.; Stuttmann, J.; Wirthmueller, L.; Betsuyaku, S.; Witte, C.P.; Bhat, R.; Pochon, N.; Colby, T, Parker, J.E. Interaction between SGT1 and cytosolic/nuclear HSC70 chaperones regulates *Arabidopsis* immune responses. *Plant Cell* **2007**, *19*, 4061–4076, doi:10.1105/tpc.107.051896.
99. Mithoe, S.; Boersema, P.; Berke, L.; Snel, B.; Heck, A.; Menke, F. Targeted quantitative phosphoproteomics approach for the detection of phospho-tyrosine signaling in plants. *J. Proteome Res*. **2012**, *11*, 438–448, doi:10.1021/pr200893k.
100. Lin, L.; Hsu, C.; Hu, C.; Ko, S.; Hsieh, H.; Huang, H.; Juan, H. Integrating Phosphoproteomics and Bioinformatics to Study Brassinosteroid-Regulated Phosphorylation Dynamics in *Arabidopsis*. *BMC Genom*. **2015**, *16*, 533, doi:10.1186/s12864-015-1753-4.
101. Sugiyama, N.; Nakagami, H.; Mochida, K.; Daudi, A.; Tomita, M.; Shirasu, K.; Ishihama, Y. Large-scale phosphorylation mapping reveals the extent of tyrosine phosphorylation in *Arabidopsis*. *Mol. Syst. Biol.* **2008**, *4*, 193, doi:10.1038/msb.2008.32.
102. Heazlewood, J.L.; Durek, P.; Hummel, J.; Selbig, J.; Weckwerth, W.; Walther, D.; Schulze, W.X. PhosPhAT: A database of phosphorylation sites in *Arabidopsis thaliana* and a plant-specific phosphorylation site predictor. *Nucleic Acids Res.* **2008**, *36*, 1015–1021, doi:10.1093/nar/gkm812.
