# Supplementary material for: The Complexity of Mitochondrial Complex IV: An Update of Cytochrome c Oxidase Biogenesis in Plants
Source: Int J Mol Sci. 2018 Feb 27;19(3):662. doi: 10.3390/ijms19030662 (PMC5877523; doi:10.3390/ijms19030662)
Supplement: Supplementary file 1 [file ijms-19-00662-s001.zip › Figure S3-2nd_ew.pdf]

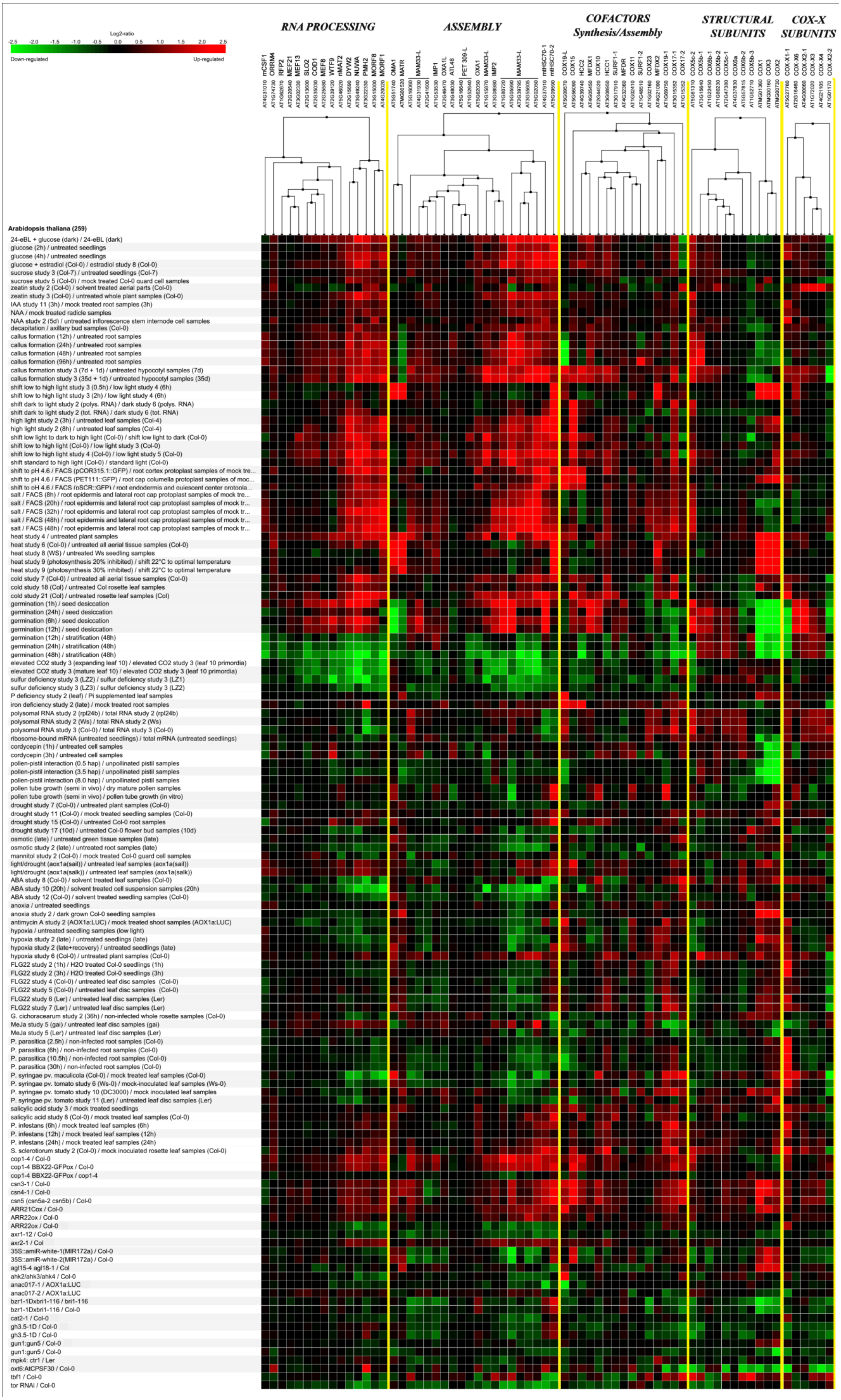
RNA PROCESSING
ASSEMBLY
COFACTORS Synthesis/Assembly
STRUCTURAL SUBUNITS
COX-X SUBUNITS
Log2-ratio
-2.5 -2.0 -1.5 -1.0 -0.5 0.0 0.5 1.0 1.5 2.0 2.5
Down-regulated
Up-regulated
mCSF1 ORRM4 RFP2 MEF21 MEF13 SLO2 COD1 MEF8 WTF9 nMAT2 DYW2 NUWA PMH2 MORF8 MORF1
OMA1 MATR MAM33-L IMP1 OXA1L ATL48 PET 309-L OXA1 MAM33-L IMP2 MAM33-L mtHSC70-1 mtHSC70-2
COX19-L COX15 HCC2 MFDX1 COX10 HCC1 SURF1-1 MFDR COX11 SURF1-2 COX23 MFDX2 COX19-1 COX17-1 COX17-2
COX5c-2 COX5b-1 COX6b-1 COX5b-2 COX5c-1 COX6a COX6b-2 COX5b-3 COX1 COX3 COX2
COX-X1-1 COX-X6 COX-X2-1 COX-X3 COX-X4 COX-X2-2
Arabidopsis thaliana (259)
24-eBL + glucose (dark) / 24-eBL (dark)
glucose (2h) / untreated seedlings
glucose (4h) / untreated seedlings
glucose + estradiol (Col-0) / estradiol study 8 (Col-0)
sucrose study 3 (Col-7) / untreated seedlings (Col-7)
sucrose study 5 (Col-0) / mock treated Col-0 guard cell samples
zeatin study 2 (Col-0) / solvent treated aerial parts (Col-0)
zeatin study 3 (Col-0) / untreated whole plant samples (Col-0)
IAA study 11 (3h) / mock treated root samples (3h)
NAA / mock treated radicle samples
NAA study 2 (5d) / untreated inflorescence stem internode cell samples
decapitation / axillary bud samples (Col-0)
callus formation (12h) / untreated root samples
callus formation (24h) / untreated root samples
callus formation (48h) / untreated root samples
callus formation (96h) / untreated root samples
callus formation study 3 (7d + 1d) / untreated hypocotyl samples (7d)
callus formation study 3 (35d + 1d) / untreated hypocotyl samples (35d)
shift low to high light study 3 (0.5h) / low light study 4 (6h)
shift low to high light study 3 (2h) / low light study 4 (6h)
shift dark to light study 2 (polys. RNA) / dark study 6 (polys. RNA)
shift dark to light study 2 (tot. RNA) / dark study 6 (tot. RNA)
high light study 2 (3h) / untreated leaf samples (Col-4)
high light study 2 (8h) / untreated leaf samples (Col-4)
shift low light to dark to high light (Col-0) / shift low light to dark (Col-0)
shift low to high light (Col-0) / low light study 3 (Col-0)
shift low to high light study 4 (Col-0) / low light study 5 (Col-0)
shift standard to high light (Col-0) / standard light (Col-0)
shift to pH 4.6 / FACS (pCOR315.1::GFP) / root cortex protoplast samples of mock tre...
shift to pH 4.6 / FACS (PET111::GFP) / root cap columella protoplast samples of moc...
shift to pH 4.6 / FACS (pSCR::GFP) / root endodermis and quiescent center protopla...
salt / FACS (8h) / root epidermis and lateral root cap protoplast samples of mock tre...
salt / FACS (20h) / root epidermis and lateral root cap protoplast samples of mock tr...
salt / FACS (32h) / root epidermis and lateral root cap protoplast samples of mock tr...
salt / FACS (48h) / root epidermis and lateral root cap protoplast samples of mock tr...
salt / FACS (48h) / root epidermis and lateral root cap protoplast samples of mock tr...
heat study 4 / untreated plant samples
heat study 6 (Col-0) / untreated all aerial tissue samples (Col-0)
heat study 8 (WS) / untreated Ws seedling samples
heat study 9 (photosynthesis 20% inhibited) / shift 22°C to optimal temperature
heat study 9 (photosynthesis 30% inhibited) / shift 22°C to optimal temperature
cold study 7 (Col-0) / untreated all aerial tissue samples (Col-0)
cold study 18 (Col) / untreated Col rosette leaf samples
cold study 21 (Col) / untreated rosette leaf samples (Col)
germination (1h) / seed desiccation
germination (24h) / seed desiccation
germination (6h) / seed desiccation
germination (12h) / seed desiccation
germination (12h) / stratification (48h)
germination (24h) / stratification (48h)
germination (48h) / stratification (48h)
elevated CO2 study 3 (expanding leaf 10) / elevated CO2 study 3 (leaf 10 primordia)
elevated CO2 study 3 (mature leaf 10) / elevated CO2 study 3 (leaf 10 primordia)
sulfur deficiency study 3 (LZ2) / sulfur deficiency study 3 (LZ1)
sulfur deficiency study 3 (LZ3) / sulfur deficiency study 3 (LZ2)
P deficiency study 2 (leaf) / Pi supplemented leaf samples
iron deficiency study 2 (late) / mock treated root samples
polysomal RNA study 2 (rpl24b) / total RNA study 2 (rpl24b)
polysomal RNA study 2 (Ws) / total RNA study 2 (Ws)
polysomal RNA study 3 (Col-0) / total RNA study 3 (Col-0)
ribosome-bound mRNA (untreated seedlings) / total mRNA (untreated seedlings)
cordycepin (1h) / untreated cell samples
cordycepin (3h) / untreated cell samples
pollen-pistil interaction (0.5 hap) / unpollinated pistil samples
pollen-pistil interaction (3.5 hap) / unpollinated pistil samples
pollen-pistil interaction (8.0 hap) / unpollinated pistil samples
pollen tube growth (semi in vivo) / dry mature pollen samples
pollen tube growth (semi in vivo) / pollen tube growth (in vitro)
drought study 7 (Col-0) / untreated plant samples (Col-0)
drought study 11 (Col-0) / mock treated seedling samples (Col-0)
drought study 15 (Col-0) / untreated Col-0 root samples
drought study 17 (10d) / untreated Col-0 flower bud samples (10d)
osmotic (late) / untreated green tissue samples (late)
osmotic study 2 (late) / untreated root samples (late)
mannitol study 2 (Col-0) / mock treated Col-0 guard cell samples
light/drought (aox1a(sail)) / untreated leaf samples (aox1a(sail))
light/drought (aox1a(salk)) / untreated leaf samples (aox1a(salk))
ABA study 8 (Col-0) / solvent treated leaf samples (Col-0)
ABA study 10 (20h) / solvent treated cell suspension samples (20h)
ABA study 12 (Col-0) / solvent treated seedling samples (Col-0)
anoxia / untreated seedlings
anoxia study 2 / dark grown Col-0 seedling samples
antimycin A study 2 (AOX1a:LUC) / mock treated shoot samples (AOX1a:LUC)
hypoxia / untreated seedling samples (low light)
hypoxia study 2 (late) / untreated seedlings (late)
hypoxia study 2 (late+recovery) / untreated seedlings (late)
hypoxia study 6 (Col-0) / untreated plant samples (Col-0)
FLG22 study 2 (1h) / H2O treated Col-0 seedlings (1h)
FLG22 study 2 (3h) / H2O treated Col-0 seedlings (3h)
FLG22 study 4 (Col-0) / untreated leaf disc samples (Col-0)
FLG22 study 5 (Col-0) / untreated leaf disc samples (Col-0)
FLG22 study 6 (Ler) / untreated leaf disc samples (Ler)
FLG22 study 7 (Ler) / untreated leaf disc samples (Ler)
G. cichoracearum study 2 (36h) / non-infected whole rosette samples (Col-0)
MeJa study 5 (gai) / untreated leaf disc samples (gai)
MeJa study 5 (Ler) / untreated leaf disc samples (Ler)
P. parasitica (2.5h) / non-infected root samples (Col-0)
P. parasitica (6h) / non-infected root samples (Col-0)
P. parasitica (10.5h) / non-infected root samples (Col-0)
P. parasitica (30h) / non-infected root samples (Col-0)
P. syringae pv. maculicola (Col-0) / mock treated leaf samples (Col-0)
P. syringae pv. tomato study 6 (Ws-0) / mock-inoculated leaf samples (Ws-0)
P. syringae pv. tomato study 10 (DC3000) / mock inoculated leaf samples
P. syringae pv. tomato study 11 (Ler) / untreated leaf disc samples (Ler)
salicylic acid study 3 / mock treated seedlings
salicylic acid study 8 (Col-0) / mock treated leaf samples (Col-0)
P. infestans (6h) / mock treated leaf samples (6h)
P. infestans (12h) / mock treated leaf samples (12h)
P. infestans (24h) / mock treated leaf samples (24h)
S. sclerotiorum study 2 (Col-0) / mock inoculated rosette leaf samples (Col-0)
cop1-4 / Col-0
cop1-4 BBX22-GFPox / Col-0
cop1-4 BBX22-GFPox / cop1-4
csn3-1 / Col-0
csn4-1 / Col-0
csn5 (csn5a-2 csn5b) / Col-0
ARR21Cox / Col-0
ARR22ox / Col-0
ARR22ox / Col-0
axr1-12 / Col-0
axr2-1 / Col
35S::amiR-white-1(MIR172a) / Col-0
35S::amiR-white-2(MIR172a) / Col-0
agl15-4 agl18-1 / Col
ahk2/ahk3/ahk4 / Col-0
anac017-1 / AOX1a:LUC
anac017-2 / AOX1a:LUC
bzr1-1Dxbri1-116 / bri1-116
bzr1-1Dxbri1-116 / Col-0
cat2-1 / Col-0
gh3.5-1D / Col-0
gh3.5-1D / Col-0
gun1:gun5 / Col-0
gun1:gun5 / Col-0
mpk4: ctr1 / Ler
oxt6:AtCPSF30 / Col-0
tbf1 / Col-0
tor RNAi / Col-0

Figure S3: Hierarchical clustering of the expression data in response to several perturbations or in different mutant backgrounds. Meta-analysis of the expression for genes encoding 68 COX-related proteins in response to different perturbations, stress conditions or in several mutant-backgrounds. Conditions where at least 30% of the genes change their expression more than 2-fold were selected. The analysis was made by exploring publicly available microarray data included in the Genevestigator database (https://genevestigator.com/gv/doc/intro_plant.jsp, [100]). Expression level is represented as log_2-ratio of differential expression, with up-regulation represented in red and down-regulation represented in green.

100. Hruz, T.; Laule, O.; Szabo, G.; Wessendorp, F.; Bleuler, S.; Oertle, L.; Widmayer, P.; Gruissem, W.; Zimmermann, P. Genevestigator V3: A reference expression database for the meta-analysis of transcriptomes. *Adv. Bioinform.* **2008**, *420747*, doi:10.1155/2008/420747.
