# Supplementary material for: The Complexity of Mitochondrial Complex IV: An Update of Cytochrome c Oxidase Biogenesis in Plants
Source: Int J Mol Sci. 2018 Feb 27;19(3):662. doi: 10.3390/ijms19030662 (PMC5877523; doi:10.3390/ijms19030662)
Supplement: Supplementary file 1 [file ijms-19-00662-s001.zip › Figure S1-2nd_ew.pdf]

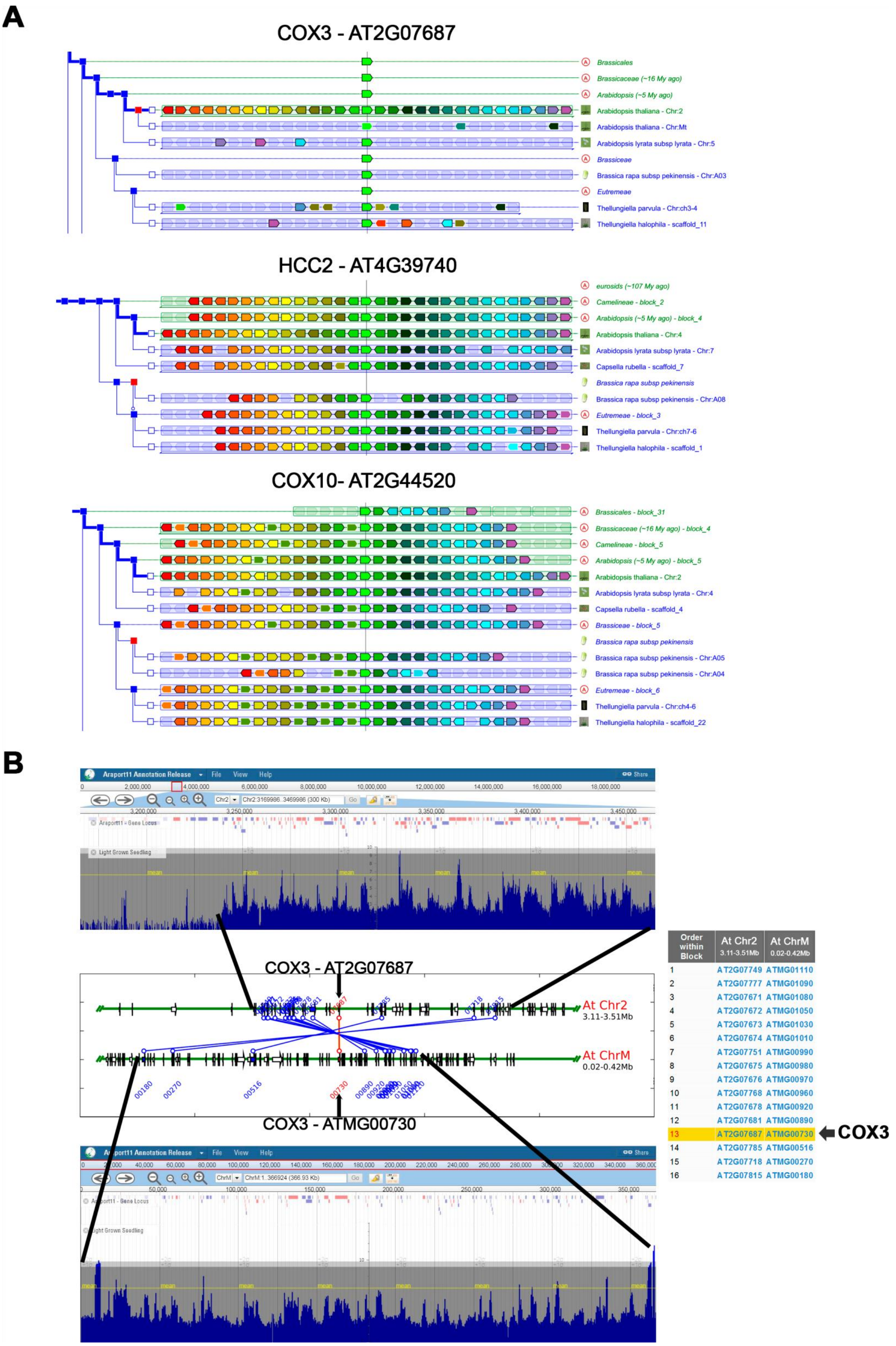

Order within Block	At Chr2 3.11-3.51Mb	At ChrM 0.02-0.42Mb
1	AT2G07749	ATMG01110
2	AT2G07777	ATMG01090
3	AT2G07671	ATMG01080
4	AT2G07672	ATMG01050
5	AT2G07673	ATMG01030
6	AT2G07674	ATMG01010
7	AT2G07751	ATMG00990
8	AT2G07675	ATMG00980
9	AT2G07676	ATMG00970
10	AT2G07768	ATMG00960
11	AT2G07678	ATMG00920
12	AT2G07681	ATMG00890
13	AT2G07687	ATMG00730
14	AT2G07785	ATMG00516
15	AT2G07718	ATMG00270
16	AT2G07815	ATMG00180

Figure S1: COX3 has an extra copy in the nuclear chromosome 2 in Arabidopsis. (A) Synteny analysis of the nuclear Arabidopsis COX3 gene in comparison with other Brassicaceae. AtCOX10 and AtHCC2 are other nuclear genes included in the analysis for comparison. **(B)** The 30 kbp region including COX3 and 15 additional genes encoding structural OXPHOS proteins or mitochondrial ribosomal proteins shows a similar arrangement in the mitochondrial and nuclear genomes. Blue lines connect similar genes. The transcriptional level of this region in both genomes, based on RNAseq data available in public databases, is shown in blue.
