# Supplementary material for: The Complexity of Mitochondrial Complex IV: An Update of Cytochrome c Oxidase Biogenesis in Plants
Source: Int J Mol Sci. 2018 Feb 27;19(3):662. doi: 10.3390/ijms19030662 (PMC5877523; doi:10.3390/ijms19030662)
Supplement: Supplementary file 1 [file ijms-19-00662-s001.zip › Figure S4-2nd_ew.pdf]

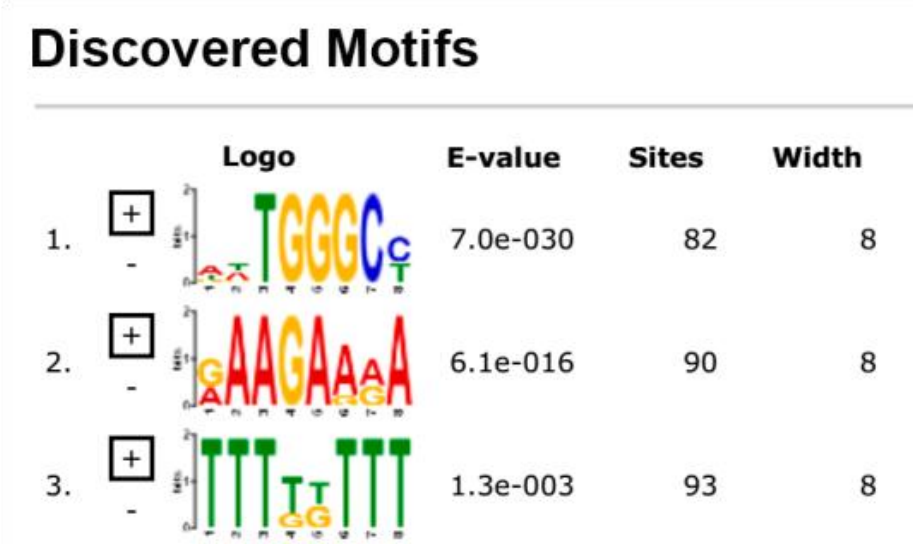

Figure S4: Common regulatory elements in the promoter regions of nuclear COX-related genes. Conserved consensus sequences overrepresented in the 500-bp region located upstream of the transcriptional start site of gene coding for COX-related proteins, identified using the MEME server [118].

118. Bailey, T.L.; Boden, M.; Buske, F.A.; Frith, M.; Grant, C.E.; Clementi, L.; Ren, J.; Li, W.W.; Noble, W.S. MEME SUITE: Tools for motif discovery and searching. *Nucleic Acids Res.* **2009**, W202–W208, doi:10.1093/nar/gkp335.
