# Supplementary material for: The Complexity of Mitochondrial Complex IV: An Update of Cytochrome c Oxidase Biogenesis in Plants
Source: Int J Mol Sci. 2018 Feb 27;19(3):662. doi: 10.3390/ijms19030662 (PMC5877523; doi:10.3390/ijms19030662)
Supplement: Supplementary file 1 [file ijms-19-00662-s001.zip › Figure S2-2nd_ew.pdf]

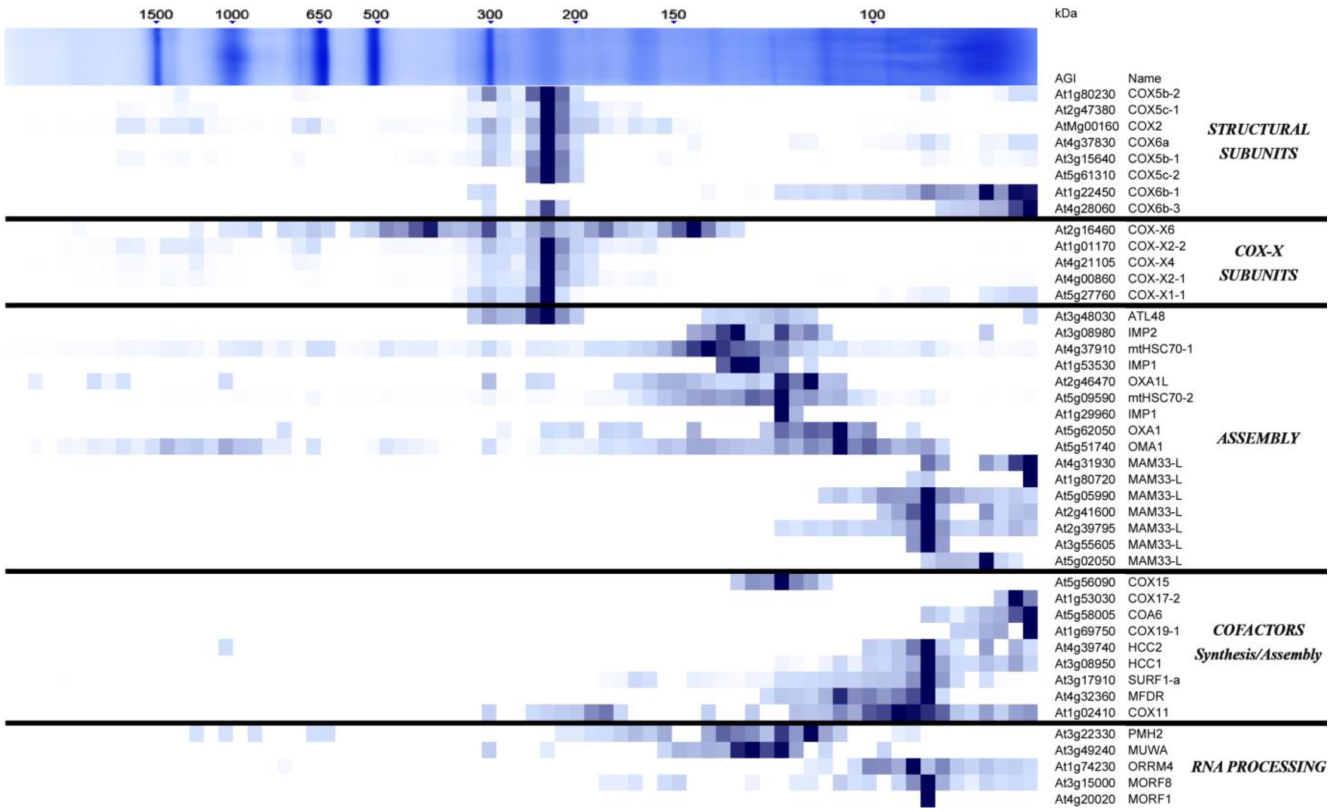

Figure S2: Migration of COX subunits and other COX-related proteins in Blue-native PAGE. Candidate proteins were classified into five different categories according to their putative or demonstrated role in COX biogenesis. Protein migration and the respective size (kDa) are shown on the top. The data were obtained from the Complexome server (https://complexomemap.de/at_mito_leaves/ [59]).

59. Senkler, J.; Senkler, M.; Eubel, H.; Hildebrandt, T.; Lengwenus, C.; Schertl, P.; Schwarzländer, M.; Wagner, S.; Wittig, I.; Braun, H.P. The mitochondrial complexome of Arabidopsis thaliana. *Plant J.* **2017**, *89*, 1079–1092, doi:10.1111/tpj.13448.
